# Heat Stress-Mediated Constraints in Maize (*Zea mays*) Production: Challenges and Solutions

**DOI:** 10.3389/fpls.2022.879366

**Published:** 2022-04-29

**Authors:** Ahmed H. El-Sappah, Shabir A. Rather, Shabir Hussain Wani, Ahmed S. Elrys, Muhammad Bilal, Qiulan Huang, Zahoor Ahmad Dar, Mohamed M. A. Elashtokhy, Nourhan Soaud, Monika Koul, Reyazul Rouf Mir, Kuan Yan, Jia Li, Khaled A. El-Tarabily, Manzar Abbas

**Affiliations:** ^1^School of Agriculture, Forestry and Food Engineering, Yibin University, Yibin, China; ^2^Department of Genetics, Faculty of Agriculture, Zagazig University, Zagazig, Egypt; ^3^Key Laboratory of Sichuan Province for Refining Sichuan Tea, Yibin, China; ^4^Center for Integrative Conservation, Xishuangbanna Tropical Botanical Garden, Chinese Academy of Sciences, Menglun, China; ^5^Mountain Research Centre for Field Crops Khudwani Anantnag, SKUAST–Kashmir, Srinagar, India; ^6^Department of Soil Science, Faculty of Agriculture, Zagazig University, Zagazig, Egypt; ^7^School of Life Sciences and Food Engineering, Huaiyin Institute of Technology, Huaian, China; ^8^College of Tea Science, Yibin University, Yibin, China; ^9^Dryland Agriculture Research Station, SKUAST–Kashmir, Srinagar, India; ^10^Department of Crop Science, Faculty of Agriculture, Zagazig University, Zagazig, Egypt; ^11^Department of Botany, Hansraj College, University of Delhi, New Delhi, India; ^12^Division of Genetics and Plant Breeding, Faculty of Agriculture (FoA), SKUAST–Kashmir, Sopore, India; ^13^Department of Biology, College of Science, United Arab Emirates University, Al Ain, United Arab Emirates; ^14^Harry Butler Institute, Murdoch University, Murdoch, WA, Australia

**Keywords:** abiotic stress, gene signaling cascade, heat stress, molecular response, *Zea mays*

## Abstract

An increase in temperature and extreme heat stress is responsible for the global reduction in maize yield. Heat stress affects the integrity of the plasma membrane functioning of mitochondria and chloroplast, which further results in the over-accumulation of reactive oxygen species. The activation of a signal cascade subsequently induces the transcription of heat shock proteins. The denaturation and accumulation of misfolded or unfolded proteins generate cell toxicity, leading to death. Therefore, developing maize cultivars with significant heat tolerance is urgently required. Despite the explored molecular mechanism underlying heat stress response in some plant species, the precise genetic engineering of maize is required to develop high heat-tolerant varieties. Several agronomic management practices, such as soil and nutrient management, plantation rate, timing, crop rotation, and irrigation, are beneficial along with the advanced molecular strategies to counter the elevated heat stress experienced by maize. This review summarizes heat stress sensing, induction of signaling cascade, symptoms, heat stress-related genes, the molecular feature of maize response, and approaches used in developing heat-tolerant maize varieties.

## Graphical Abstract

This review summarized heat stress-mediated morphological and physiological changes in maize and elucidated the molecular mechanisms responsible for maize response to heat stress. Furthermore, plausible approaches to dissecting the regulatory network associated with heat stress response and improving maize adaptation to global warming have been discussed. This figure was made using BioRender.

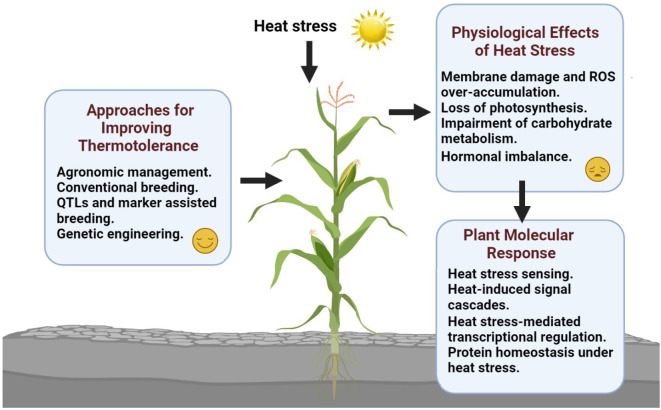

## Introduction

**FIGURE 1:**
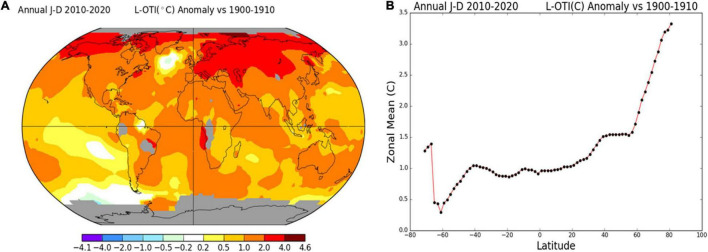
**(A)** Map of annual mean temperature change (°C) during 1900–1910 and 2010–2020. **(B)** The zonal means plot. Air temperature data of the land surface was retrieved from GHCNv4 (GISS analysis based on global historical climatology network v4), and sea surface temperature data was retrieved from ERSST_v5 (NOAA/NCEI’s extended reconstructed sea surface temperature v5). The number at the top right-hand corner of the map plot is an estimate (°C) of the global mean of the calculated area. The maps were made using GISS Surface Temperature Analysis software (https://data.giss.nasa.gov/gistemp/maps/index.html).

Heat stress is the most devastating abiotic stress factor influencing seasonal growth and spatial variations in various crops (Sallam et al., 2018; Magaña Ugarte et al., 2019). Global warming caused by the increasing growth of the population and the accompanying industrial development has become a concern that cannot be overlooked (Baus, 2017). Also, the average rise in global temperature between 1900 and 2020 was 1.13°C, and it is expected to increase by 1.4–5.8°C in 2100

(Figure 1; Houghton et al., 2001). This gradual increase in global warming and heat waves have become a serious threat to crop productivity (Hoegh-Guldberg et al., 2019). Data published by the Food and Agriculture Organization has revealed the annual relative yield loss in major cereal crops (Faostat, 2019). Also, recent studies have shown that effective heat stress tolerance *via* genetic improvement is the only possible remedy; otherwise, every 1°C temperature rise will cause a 6.0% yield loss of wheat, 3.2% of rice, 7.4% of maize, and 3.1% of soybean (Zhao et al., 2017; Kraus et al., 2022). However, due to increasing population growth, crop yield ought to increase by 70% for sustaining food security to meet the demand of a projected 9 billion population rise in 2050 (Popp et al., 2013; Dawson et al., 2016).

Maize (*Zea mays*) is an important cereal crop that belongs to the Poaceae family (Li et al., 2022) and has ensured global food security with a worldwide production ≥1 × 10^9^ t (10^12^ kg) since 2013 (Faostat, 2017). Maize was initially cultivated in tropical areas under rainfed conditions (Li J. et al., 2021; Maitra et al., 2021). However, there is an increased demand for maize due to its utilization of carbohydrates as biomass for ethanol fuel production, leaves and stem as livestock fodder, grains as raw material in the baking industry, and food and feed crop in many countries (Rooney et al., 2007; Parmar et al., 2017; Dar et al., 2021). Maize is a rich source of starch and calcium in addition to numerous essential minerals, vitamins, and fiber. However, it labors to some nutrients, such as vitamins B12 and C (McKevith, 2004). Iron absorption, particularly the non-heme iron present in maize, can be inhibited by some components of the diet being consumed, such as vegetables, coffee (e.g., polyphenols), tea (e.g., oxalates), milk (e.g., calcium), and eggs (e.g., phosvitin) (Ranum et al., 2014).

Elevated temperature accelerates crop growth but shortens its growing season (Mo et al., 2016; Hu et al., 2017; Ahmed et al., 2018; Ihsan et al., 2019). Additionally, maize growth requires an optimum daytime temperature range of 28–32°C, comparatively higher than the optimum temperature necessary for other cereal crops, such as wheat (*Triticum aestivum*) and rice (*Oryza sativa*) (Sánchez et al., 2014).

The global change resulting from harsh climatic conditions has negatively affected maize crop yields (Lobell et al., 2011; Ahmed et al., 2018; El-Sappah and Rather(eds)., 2022). Also, increased temperature stimulates the over-accumulation of phenolic compounds, resulting in cell necrosis, consequently contributing to maize yield loss (Tebaldi and Lobell, 2018). Furthermore, heat stress (>32°C) causes the deterioration of several metabolic processes in maize plants, including a severe break in photosynthesis, increased surface transpiration rate (Crafts-Brandner and Salvucci, 2002; Sharma et al., 2020), pollen-sterilization at anthesis (flowering stage) (Gourdji et al., 2013), kernels shortening at grain-filling stage (Singletary et al., 1994; Rezaei et al., 2015), cumulatively resulting in a significant yield loss.

The approval of multiple agronomic and breeding alternatives along with advanced genomic tools is inevitable to cope with the deleterious effects of extreme temperatures (Waqas et al., 2021). Several agronomic management practices, such as the management of soil and nutrients, crop rotation, plantation rate, timing, and irrigation, are beneficial for the development of heat tolerance in maize (Sabagh et al., 2020). Genetically modified crops could also be a valuable resource for the development of novel traits that enhance the survival of plants under harsh conditions (Jha et al., 2020). In recent years, the rate of crop improvement has accelerated owing to the rapid progress in plant molecular biology. In several crops, different genetic approaches, including marker-assisted selection (MAS), map-based gene cloning, quantitative trait locus (QTL) mapping, and genome editing (such as RNA interference [RNAi] and CRISPR)/CRISPR-associated-9, Cas9), have been utilized for the selection and improvement of plant traits (Waqas et al., 2021).

This review summarized heat stress-mediated morphological and physiological changes in maize and elucidated the molecular mechanisms responsible for maize response to heat stress. We also discussed plausible approaches in dissecting the regulatory network associated with heat stress response and improving maize adaptation to global warming.

## Impact of Heat Stress on Different Growth Stages

### Vegetative Stage

**FIGURE 2:**
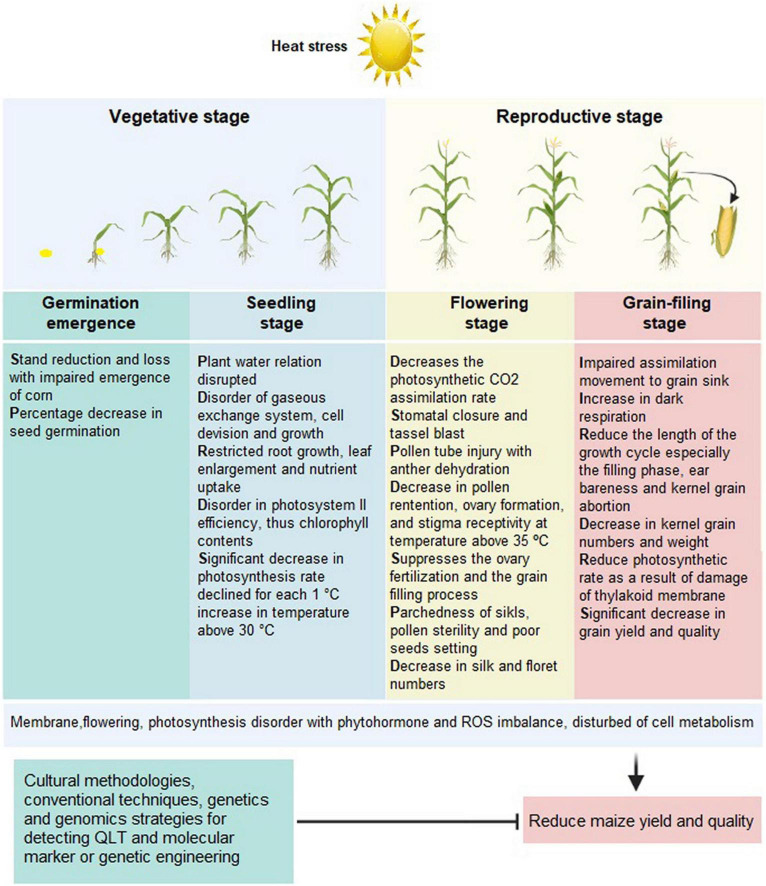
Morphological and physiological characteristics of maize under heat stress. This figure was made using BioRender.

Technically, the growth of stems, leaves, and roots, usually referred to as vegetative growth, is also known as germination, leaf, and tasseling (Dolatabadian et al., 2010). Heat stress affects the abovementioned growth stages (Figure 2) significantly. Also, the optimum soil temperature for maize seeds germination is 21°C, whereas <13°C causes a severe stoppage in germination and <10°C causes a total cessation (Kaspar and Bland, 1992; Towil, 2010; Sánchez et al., 2014). The germination rate of spring sowing of maize seeds cultivated in higher altitudes, such as North Europe and North America, is comparatively low due to low soil temperature (Paul et al., 1996). Early seed germination may expose the crop to freezing temperature, and early flowering leads to short crop duration leading to severe yield loss (Jagadish et al., 2016). However, late cultivation for optimum temperature conditions caused a severe loss in yield due to pest attacks (Rosenzweig et al., 2001). Therefore, only the day-neutral spring maize is favorably cultivated in higher altitudes (Colasanti and Muszynski, 2009).

Notably, the younger seedlings are less susceptible to high temperatures (Sánchez et al., 2014). The overall required temperature range for early maize seedling growth is 30–35°C, and the optimal temperature is around 20°C (Khaeim et al., 2022), 4–6°C higher than the suitable temperature for wheat and barley growth (Sánchez et al., 2014). Importantly, depending upon maize variety and below 20°C, every 0.5°C downfalls in daily temperature resulted in 10–20 days extended crop duration (Rahman et al., 2009). At an average daytime temperature of 15°C may take 200 days for the maturity of maize crop (Wilson et al., 1995).

Maize is susceptible to cold temperature but can recover from its effects if height is less than 15 cm when exposed to cold (Sakai and Larcher, 2012). Temperature below 10°C causes stunted root growth, whereas 17°C temperature results in 1.5 mm root growth per day, and temperature above 40°C inhibits root growth (Ryel et al., 2002). Maize seedlings can recover from constraints of drought stress because it is naturally resistant to drought (Daryanto et al., 2016). In conclusion, maize can recover from adverse climatic conditions if exposed at very early vegetative growth stages. The early cultivation of maize also facilitates the avoidance of pest attacks and the possible development of diseases (Bruns, 2003). So, early sowing of maize is highly recommended.

### Reproductive Stage

The fruit setting stage is the reproductive stage that begins with vegetative growth termination and flowering initiation. The stage is susceptible to unexpected fluctuation in temperature, i.e., >32°C temperature, or frost causing severe yield loss (Silim et al., 2006; Siebers et al., 2017). Also, hailstorm adversely affects outcomes at the jointing and silking stage (Chen K. et al., 2018). Similarly, soil moisture contents before, during, and after silking result in a severe reduction in yield by 25, 21, and 50%, respectively (Pandey et al., 2000). The optimum temperature at tasseling is between 21 and 30°C (Kiniry and Bonhomme, 1991). Additionally, elevated temperature encourages respiration (Guo et al., 2019) and shortens grain-filling duration, contributing to a significant yield loss (Sánchez et al., 2014). Conversely, low-temperature extends the length of the grain-filling period, the appropriate phase change of photosynthesis to dry matter, and grain filling, resulting in a higher yield (Dordas, 2009; Edreira et al., 2014; Chao et al., 2016). Overall, during pollination and grain filling, temperatures ≥35°C suppress fertilization in maize and decreases its yield by 101 kg/ha per day (Naveed et al., 2014; Dawood et al., 2020).

## Physiological Effects of Heat Stress

### Membrane Damage and Reactive Oxygen Species Over-Accumulation

Heat stress causes cell physiological changes, such as inactivating the photosystem II (PSII) reaction center and the denaturation of the lipid bilayer and embedded proteins in the thylakoid membrane, resulting in the damaging of the cell membrane (Yang et al., 1996; Nijabat et al., 2020). The damaged cell membrane has caused severe retardation of ion exchange, leakage of electrolytes, viscous cytosol due to water loss, toxic compounds production, and homeostasis disruption (Stanley and Parkin, 1991; Demidchik, 2015). Also, these changes have resulted in plant growth cessation through leaf wilt, reduced leaf area, and leaf abscission (Bartels and Sunkar, 2005; Mafakheri et al., 2010). Furthermore, the cell membrane stability varies with plant tissue age, growth stage, growing season, plant species, and heat intensity (Nijabat et al., 2020). Therefore, the plant’s retention of its cell membrane stability and water contents under heat stress during the vegetative and reproductive growth period has generated higher yields (Khakwani et al., 2012).

Heat stress stimulates ROS biosynthesis that promotes membranous lipids peroxidation, leakage of cellular contents, protein degradation, enzymatic inactivation, bleaching of chlorophyll pigments, and DNA damage, consequently resulting in necrosis (He and Häder, 2002; Mujahid et al., 2007). Phospholipids-peroxidation causes the production of malondialdehyde (MDA) which causes damage to the cell membrane (Pamplona, 2008; Wadhwa et al., 2012). Additionally, ROS causes polyunsaturated fatty acid peroxidation, leading to chain breakage contributing to increased membrane permeability and fluidity (Catalá, 2009). Notably, the increased accumulation of H_2_O_2_ causes lipid peroxidation and membrane damage (Banerjee and Roychoudhury, 2018; Yadav et al., 2018). Heat stress-mediated genetic variations have been investigated in several cereal crops, including wheat, barley, rice, and maize (Kumari et al., 2009; Khajuria et al., 2016; Swapna and Shylaraj, 2017). Balanced redox reaction system activation via enzymatic antioxidants, such as superoxide dismutase, catalase, ascorbate peroxidase, glutathione reductase, and non-enzymatic antioxidants, such as NADH; NADPH; ascorbic acid, glutathione, and secondary metabolites play a crucial role in heat stress tolerance (Wahid et al., 2007; Foyer and Shigeoka, 2011).

### Loss of Photosynthesis

Photosynthetic apparatus is highly vulnerable to damage when exposed to heat stress and intense light (Essemine et al., 2012; Li Y. T. et al., 2020). Therefore, heat stress causes a severe reduction in carbon assimilation, restricts electron transfer, aggravates oxidative damage and photoinhibition of PSII, resulting in significant yield loss (Elferjani and Soolanayakanahally, 2018; Li Y. T. et al., 2020). Heat stress also denatures vital enzymes associated with the Calvin cycle, such as rubisco, and reduces carbon assimilation in C3 plants (Dias and Brüggemann, 2010; Zhang et al., 2020a). However, C4 plants, such as maize, harbor the CO_2_ concentration mechanism (Dai et al., 1993; Majeran and van Wijk, 2009), reducing the restriction of photosynthetic carbon assimilation *via* the Calvin cycle (von Caemmerer and Furbank, 2016). Furthermore, Phosphoenol pyruvate carboxylase is the highly thermostable initial enzyme involved in the C4 cycle (O’Leary et al., 2011), suggesting that other photosynthesis pathways contribute to declining photosynthetic carbon assimilation under heat stress in maize (Li Y. T. et al., 2020). Notably, the photosynthetic apparatus acclimatizes to heat stress by improving its antioxidant capacity and changing leaf structure and metabolism (Li Y. T. et al., 2020). However, shock heat stress during flowering causes irreparable yield loss by damaging the leaves, rendering them unable to sprout again due to the completion of vegetative growth (Li Y. T. et al., 2020).

Respiration plays a crucial role in photosynthesis, whereas its inhibition suppresses CO_2_ fixation and photoinhibition (Gardeström and Igamberdiev, 2016). However, stomatal closure does not limit the exchange of gases like CO_2_ but limits the transpiration rate through leaves. The CO_2_ concentration mechanism of C4 plants, such as maize leaves, provides more robust resistance to stomatal restriction than in C3 plants (Markelz et al., 2011). Additionally, the blockage of respiratory electron transfer inhibits photorespiration resulting in PSII photoinhibition (Rochaix, 2011; Zhang et al., 2017). Transpiration through stomata is an important heat-dissipating mechanism, with their closure under heat stress resulting in severe loss in net photosynthetic rate (Pn) (Caine et al., 2019). The lower stomatal conductance (Gs) in maize leaves maintains water-use efficiency but damages photosynthetic apparatus under heat stress. Therefore, the lower Gs due to stomata closure indicates less heat dissipation *via* the transpiration mechanisms in the leaves of C4 plants, such as maize, compared to C3 plants (El-Sharkawy, 2007; Li Y. T. et al., 2020).

Photoinhibition of photosystems (PSI and PSII) in the chloroplast results from the degradation of the light receptors under heat stress contributing to the significant halt in photosynthesis (Zivcak et al., 2015). The oxygen-evolving complex (OEC) of PSII is highly sensitive to heat stress than of high-intensity light, whereas the D1 protein of PSII is more sensitive to high-intensity light instead of heat stress (Vass and Cser, 2009; Tóth et al., 2011). It is reported that heat stress significantly affects the acceptor site of PSII instead of PSI in maize leaves (Yan et al., 2013; Li Y. T. et al., 2020). Accordingly, OEC is the primary site in maize leaf cells affected by heat stress, whereas D1 is the primary site affected by high-intensity light. The over-accumulation of ROS is another cause of D1 protein denaturation (Kong et al., 2013). Therefore, overexpression of OEC and D1 protein and downregulation of ROS *via* genetic engineering and breeding techniques will improve heat tolerance in maize (Li and Howell, 2021).

### Imbalance/Deregulation in Primary and Secondary Metabolism

Traditionally, metabolites are divided into primary and secondary/specialized metabolites. Primary metabolites reinforce cell and secondary/specialized metabolites are concerned with an organism’s interaction with its environment. Primary metabolism produces precursors for secondary metabolite biosynthesis and plays a direct and central role in plant growth, development, and reproduction. It also produces precursors for secondary metabolite biosynthesis (Medeiros et al., 2021). Secondary metabolites possess functional and chemical diversity (Erb and Kliebenstein, 2020). Thousands of metabolites serve as mediators for the various interactions between plant and the environment (Medeiros et al., 2021). During a stress response, plants fine-tune their metabolic production accordingly; however, the mechanisms, reasons, and regulations for this process are only partially understood.

Leaf metabolites were most affected by long-duration salt, heat, or drought stress treatments compared with the rest of the maize organs. The raffinose pathway metabolites (raffinose and galactinol) and some amino acids such as threonine, tryptophan, and histidine also stood out in the heat stress metabolome profile (Joshi et al., 2021). In the metabolic studies of Joshi et al. (2021), 2,549 genes were upregulated including galactinol synthase (*Zm00001d028931*), stachyose synthase (*Zm00001d039685*), and a putative inositol transporter (*Zm00001d018803*), while 2,587 genes were downregulated as a result of heat stress. Two stress-induced arginine decarboxylase paralogs exhibited a similar dichotomy with drought and heat, inducing *Zm00001d051194*. However, the responses from pairing drought and heat stressors contrasts with the pattern exhibited by the raffinose pathway genes described above where the effects of heat and salt were correlated (Joshi et al., 2021).

Heat stress adversely affects carbohydrate catabolism by denaturing relevant enzymes resulting in the over-accumulation of starch and sucrose (Ruan et al., 2010; Xalxo et al., 2020). Varied expression patterns of genes and proteins involved in carbohydrate metabolism were observed in *Arabidopsis* exposed to heat stress (Kaplan et al., 2004). In addition, heat stress causes over-accumulation of maltose, sucrose, and cell wall-specific monosaccharides (Lima et al., 2013; Sengupta et al., 2015). Additionally, the metabolic profiling of plants exposed to two abiotic stress factors, such as drought and heat, showed over-accumulation of glucose, fructose, sucrose, trehalose, maltose responsible for maintaining cell turgor pressure, stabilizing cell membranes and proteins (Rodziewicz et al., 2014; Kumar et al., 2021). During unfavorable conditions, plants digest starch molecules to get energy as a substitute for glucose; however, extended heat stress causes depletion of all carbohydrate reservoirs and causes plants starvation (Kaplan et al., 2004; Djanaguiraman et al., 2010).

Temperature significantly affects starch biosynthesis in maize kernels, which contributes to the total dry weight of grains (Keeling et al., 1994). Heat stress stimulates the production of osmolytes including fructose, mannose, sucrose, and proline, which plays a vital role in heat stress tolerance (Slama et al., 2015; Sharma et al., 2019). The grain-filling rate and duration are determined by the sucrose contents available in kernels and enzyme activity level (Singletary et al., 1994; Alam et al., 2021). Short interval time series analysis revealed that the “tipping point” for maize metabolome perturbation is lengthened at a >1 day of drought stress, including a combined effect of drought and heat stress (Bechtold et al., 2016). Generally, heat stress causes mechanical changes, whereas drought stress results in the disequilibrium of osmosis in plants cell (Haswell and Verslues, 2015). Therefore, abiotic stress-mediated changes in metabolic responses are probably attributed to adaptations to drought and heat stresses (Kaplan et al., 2004; Khan et al., 2015).

Osmolytes also contribute a crucial role in maintaining membrane structure (Sharma et al., 2019), alleviating proteins degradation, reducing ionic toxicity, protecting cell organelles, scavenging ROS, protecting antioxidant compounds, and maintaining redox equilibrium (Hasanuzzaman et al., 2020). Osmolytes, such as sucrose, fructose, and mannose, are resources of energy, nutrition, structural materials, signaling molecules, and crucially contribute to seed germination and the growth of plantlets (Osuna et al., 2015). Maize (*Zea mays* L.) seedlings exposed to heat stress displayed sudden degradation of glycan contents and upregulated fructose and mannose metabolism (Lieu et al., 2021). The expression of genes involved in fructose, mannose, and sucrose biosynthesis was also upregulated in 21-day-old maize seedlings exposed to heat stress (Stavridou et al., 2021).

The mitochondria and nuclear membrane structure were also disrupted by heat stress, more severe in the heat-sensitive hybrid (Török et al., 2014; Li Y. T. et al., 2020). Also, disruption of mitochondrial membrane structure decreases the efficiency of oxidative phosphorylation, requiring increased consumption of carbohydrates to supply sufficient ATP and further reducing light energy utilization (Li Y. T. et al., 2020). Additionally, many chloroplast proteins are encoded by the nuclear genome; hence, destruction of the nuclear envelope may inhibit the upregulation of photo-protection mechanisms, aggravating the photosynthetic mechanism damage and delaying photo inhibition repair and structural damage (Kumar and Kaushik, 2021). The less grouped PSII units are more sensitive to light, partly explaining the more severe PSII under heat stress (Strasser et al., 2004).

### Hormonal Imbalance

Phytohormones, such as auxin/indole acetic acid (IAA), gibberellic acid (GA), abscisic acid (ABA), cytokinin (CTK), ethylene (ET), salicylic acid (SA), brassinosteroids (BRs), strigolactone (SL), and jasmonic acid (JA) importantly regulates cellular processes which are ubiquitous to plant growth under abiotic stress factors (Sharma et al., 2019). Heat stress causes over-accumulation of ABA and the downregulation of CTK, resulting in the improper development of maize kernels (Cheikh and Jones, 1994; Niu et al., 2021). The application of benzyladenine on maize seedlings maintains a proper balance between ABA and CTK, causing an increased heat tolerance (Cheikh and Jones, 1994). Similarly, the treatment of maize seedlings with Ca^2+^ ions solution and ABA improves the antioxidant enzyme activity, reduces lipid peroxidation, and improves heat tolerance (Hossain et al., 2015; Yang et al., 2021). Similarly, SA, GA, and H_2_S stimulate the biosynthesis of proline, betaine, and trehalose, contributing to the enhanced antioxidant activity in maize (Li, 2015; Li Z. G. et al., 2015; Zhou et al., 2018). Overexpression of *ZmbZIP4* induces longer primary roots, more lateral roots, and enhanced biosynthesis of ABA, which cumulatively results in enhanced abiotic stress tolerance (Ma et al., 2018).

## Molecular Mechanisms of Plant Responses to Heat Stress

### Heat Stress Sensing

**FIGURE 3:**
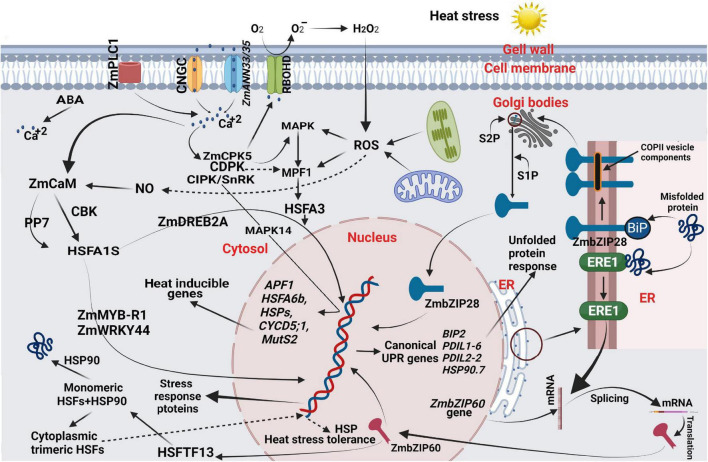
A heat stresses responsive regulatory network in maize. Heat stress damages the cell membrane when exposed to heat stress and promotes the release of apoplastic Ca^2+^. Heat stress disturbs the plasma membrane’s fluidity and permeability, resulting in a loss in function of chloroplasts and mitochondria, higher cytosolic Ca^2+^, ROS, NO, and over-accumulation of misfolded or unfolded proteins. Increased cytosolic Ca^2+^, ROS, and NO are secondary messengers and stimulate downstream regulatory networks. Heat stress disrupts protein homeostasis, inducing unfolded-protein response (UPR) and signaling pathways mediated by IRE1-*ZmZIP60* and *ZmZIP28*. The *ZmZIP60* activates the expression of a type-A HSF and *HSFTF13*, which upregulates the expression of HSP genes, i.e., *Hsp90*. The Ca^2+^ signaling is essential in heat tolerance of seed-set in maize under field conditions, where calcium, a critical secondary messenger, converges signals transmitted from high temperature, membrane fluidity, calcium efflux, and ABA (among others), amplifying them through the activation downstream of genes, such as *HSFA6b*, *ABF1*, *CYCD5*;1, *MutS2*, and HSPs during reproductive stage via the MAPK pathway, and eventually enhance maize tolerance to heat stress for seed-set. This figure was made using BioRender.

Plant cells and organelles harbor an efficient heat sensing mechanism that subsequently stimulates a signaling cascade for rapid adaptive modifications (Figure 3; Nievola et al., 2017; Niu and Xiang, 2018). For example, calcium ions (Ca^2+^) flow through their conducting channels, acting as messengers in a signaling cascade to sense and respond to heat stimuli (Jammes et al., 2011). The plasma membrane is also a primary heat-sensing organelle that contains three types of Ca^2+^ conducting channels, including voltage-dependent Ca^2+^-permeable channels (VDCCs), voltage-independent Ca^2+^-permeable channels (VICCs), depolarization-activated Ca^2+^-permeable channels (DACCs), and hyperpolarization-activated Ca^2+^-permeable channels (HACCs) (Horváth et al., 2012; Liu et al., 2018). These channels are also known as cyclic nucleotide-gated ion channels (CNGCs), naturally tetrameric cationic, and comprise six transmembrane domains (Urquhart et al., 2011). Notably these CNGCs can be genetically modified as homotetrameric or heterotetrameric to improve their ability to respond to diverse and variable intensities (Ketehouli et al., 2019; Tan et al., 2020).

In maize, 11 plasma membrane-localized CNGC genes were identified, contributing a major role in heat tolerance (Hao and Qiao, 2018). The downregulation of *AtCNGC2*, *AtCNGC6*, *PpCNGCb*, and *PpCNGC* resulted in an increased accumulation of the following heat shock proteins; *Hsp18.2*, *Hsp25.3*, and *Hsp70* (Gao et al., 2012; Finka and Goloubinoff, 2014). Glutamate heat receptor-like channels also stimulated the Ca^2+^ signaling cascade on exposure to heat stress, and the exogenous application of glutamate resulted in improved heat tolerance in maize (Li et al., 2019). Other calcium channel families have been identified as responsible for the heat tolerance capability in maize, such as downregulation of synaptotagmin A that caused the downregulation of HSPs (Yan et al., 2017; Bourgine and Guihur, 2021). Under heat stress, maize annexin, such as *AnxZm33* and *AnxZm35* expression stimulated HACCs in the roots and cytosol (Bassani et al., 2004; Nichols, 2005; Mortimer et al., 2008; He et al., 2019). Phosphoinositide-specific phospholipases C (PLCs) genes, such as *PLC3* and *PLC9* are plasma membrane-localized heat sensors that stimulate phosphoinositide-signaling mediated Ca^2+^ channels (Rupwate and Rajasekharan, 2012; Hayes et al., 2021). For example, *ZmPLC1* encodes a PI-PLC, which plays a major role in maize roots during drought stress (Zhai et al., 2013).

Additionally, heat stress alters the normal working of the chloroplasts and mitochondria membranes, resulting in the over-accumulation of ROS simultaneously stimulating the Ca^2+^ signaling pathway (Li B. et al., 2018; Navarro et al., 2021). ROS, including NADPH-oxidase (NOX) and respiratory burst oxidase homolog, also stimulate signaling cascade for heat tolerance (Sagi and Fluhr, 2006; Takemoto et al., 2007; Chapman et al., 2019). However, the over-accumulation of ROS stimulates a Ca^2+^ based signaling cascade in the cytosol, which then stimulates phosphorylation mediated calcium-dependent protein kinases (CDPKs), causing a direct activation of the respiratory burst oxidase homolog D (RBOHD) (Gao et al., 2014; Marcec et al., 2019). RTH5 family proteins comprise four transmembrane functional domains responsible for membrane embedding and two EF motifs, FAD and NAD, required for Ca^2+^transport (Lin et al., 2009; Nestler et al., 2014). In maize, RTH5 protein encodes NOX, distributed among all eukaryotic species (Bedard et al., 2007).

### Heat-Induced Signal Cascades

Heat-sensitive CNGC gene families comprise the cyclic nucleotide-binding domain and calmodulin-binding domain (CaMBD), facing toward cytosol (Gao et al., 2012; Duszyn et al., 2019). Ca^2+^ sensor-dependent transcription regulation depends upon calcineurin b-like protein (CBL), CDPK, and calmodulin (CaM) (Reddy et al., 2004, 2011; Hashimoto and Kudla, 2011). CDPKs can sense Ca^2+^ to assist their EF-hand domain and transduce Ca^2+^ signals *via* their protein kinase domain (Shi et al., 2018). In maize, 35 CDPKs were identified (Ma et al., 2013), and *ZmCDPK1* has been characterized in cold-stressed roots and leaves (Weckwerth et al., 2015). CaMs bind with the C-terminal of CNGC family genes to activate the heat shock signaling pathway (Hao and Qiao, 2018), as mitogen-activated protein kinase 6 and calmodulin-binding protein kinase 3 (CBK3) (Yan et al., 2017). In maize, the Ca^2+^–CaM contributes to the activation of ABA-induced antioxidants and nitric oxide (NO) production (Hu et al., 2007; Sang et al., 2008).

Many TFs, such as bZIP, CAMTA, MYB, and WRKY, bind with CaM proteins due to various abiotic and biotic stresses effects (Table 1; Yang et al., 2013). Among all, the CAMTA-mediated transcriptional regulation network is dominant, contributing against the diverse environmental stresses, including heat stress, salinity, drought, heavy metals, and exogenous application of hormones (Pandey et al., 2013; Yang et al., 2013; Yue et al., 2015). Additionally, CAMTA genes also play a key role in the mutual induction of regulation in expressing different stress-responsive genes and hormones (Reddy et al., 2000; Yang and Poovaiah, 2002). For example, heat stress induces upregulation of multiple *ZmCAMTA* genes in maize plants (Atkinson et al., 2013). In maize, *ZmCAMTA1*, *ZmCAMTA2*, and *ZmCAMTA3* have been identified, and their expression was upregulated during heat stress (Yue et al., 2015).

**TABLE 1:** Heat stress-related transcription factor (TF) families in maize.

Family	Gene	Function/stress	References
HSF	*ZmHsf-01*	Heat stress response The upregulation of *ZmHsf-01* is probably with H3K9 hyperacetylation in the promoter region after heat treatment	Lin et al., 2011; Kim et al., 2012; Zhang et al., 2020c
	*ZmHsf-03*	Heat stress response	Lin et al., 2011
	*ZmHsf-04*	Heat stress response	Lin et al., 2011
	*ZmHsf05*	Heat stress response	Jiang et al., 2017
	*ZmHsf06*	Heat stress response	Li H.-C. et al., 2015
	*HSFA6b*	Heat stress response Connects ABA signaling and ABA-mediated heat responses	Huang et al., 2016; Gao et al., 2019
	*HSFA1*	Stimulates immediate expression of different heat shock responsive transcription factors (TFs), including *DREB2A*, *HSFA2*, *HSFA7*, HSFBs, and multiprotein-bridging factor 1C (*MBF1C*)	Yan et al., 2020; Zhao J. et al., 2021
	*Hsftf13*	Responses to ABA And thermotolerance Activate the Hsp90 and other HSFs	Huang et al., 2016; Li Z. et al., 2020
	*ZmHsf-11*	Heat stress response	Lin et al., 2011
	*ZmHsf-17*	Heat stress response	Lin et al., 2011
	*ZmHsf-23*	Heat stress response	Lin et al., 2011
	*ZmHsf-25*	Heat stress response	Lin et al., 2011
DREB/CBF	*ZmDREB2A*	Salt, heat, drought, and cold	Qin et al., 2007
AP2/EREBP	*Zm00001d008546*	Heat stress response	Jagtap et al., 2020
MYB/MYC	*ZmMYB-R1*	Cold, salinity, drought, ABA, and heat	Liu et al., 2012
bZIP	*ZmbZIP60 (Zm00001d046718)*	Heat stress bzip28 and bzip60 double-mutant plants are sensitive to heat stress Activates the expression of a type-A HSF, Hsftf13, which, in turn, upregulates the expression of a constellation of HSP genes	Liu et al., 2012; Li Z. et al., 2020
	*ZmbZIP17*	Drought, ABA, heat, and salt	Jia et al., 2009
	*ZmbZIP28*	Encodes an ER membrane-associated bZIP transcription factor, contributes to the upregulation of heat-responsive genes and to heat tolerance *bZIP28* binds directly to the promoters of heat-responsive genes	Gao et al., 2008; Zhang et al., 2017
	*ZmbZIP4*	Heat, cold, salinity, and ABA Contributes to stress resistance in maize by regulating ABA synthesis and root development	Ma et al., 2018
NAC	*Zm00001d010227*	Drought and heat stress	Jagtap et al., 2020
GARP	*Zm00001d044785 (ZmGlk1)*	Heat stress The expression of *ZmGLK1* or *ZmG2* in rice leads to elevated levels of Chl, carotenoid, and xanthophyll cycle pigments and to increased levels of some PSII components	Jagtap et al., 2020; Yeh et al., 2021
WRKY	*ZmWRKY44*	Salt, heat, ABA, and H_2_O_2_ Have transcriptional activation functions	Kimotho et al., 2019
	*ZmWRKY106*	Drought, high temperature, ABA, and salt Play a role in the abiotic stress response by regulating stress-related genes through the ABA-signaling pathway Reactive oxygen species (ROS) scavenging	Wang et al., 2018a
	*ZmWRKY40*	Drought, salinity, heat, and ABA Regulating stress-responsive genes, such as *DREB2B* and *RD29A*	Wang et al., 2018b; Leng and Zhao, 2020
HSP	*ZmERD2*	Heat, salinity, cold, PEG, and dehydration	Song et al., 2016
	*ZmERD3*	mRNA accumulation	Song et al., 2018
NF-Y	*ZmNF-YA3*	Drought and heat *ZmNF-YA3* is directly bound to the promoter regions of two bHLH TFs (*bHLH92* and FMA) and one bZIP TF (*bZIP45*) involved in the ABA-related pathway	Su et al., 2018; Kimotho et al., 2019

Heat stress affects plasma membrane, mitochondria, endoplasmic reticulum, and chloroplasts, resulting in ROS over-accumulation, a critical secondary signaling messenger (Sewelam et al., 2014; Czarnocka and Karpińskiski, 2018).

When maize was exposed to high temperature, the related genes for protein processing in the endoplasmic reticulum (ER) pathway were significantly enriched, which mainly induced heat shock proteins expressions, such as *Hsp40*, *Hsp70*, *Hsp90*, *Hsp100* (Table 2), and small HSP (Qian et al., 2019). In response, heat stress response (HSR) genes, such as MYB, AP2/EREBP, NAC, BRs, HSPs, Rubisco, antioxidants (APX and Glutathione *S*-transferase), and kinases are activated to respond to ROS (Khan et al., 2019; Jagtap et al., 2020). ROS, such as H_2_O_2_ produced by RBOHD, acted as a signaling molecule that directly stimulates mitogen-activated protein kinases, such as MAPK3 and MAPK6, which activate Ca^2+^ or CDPK-mediated phosphorylation *HSFA2* and *HSFA4a* (Luna et al., 2011; Frederickson Matika and Loake, 2014). H_2_O_2_ also directly activates *HSFA1a*, *HSFA4a*, and *HSFA8* transcription factors, and NO signaling cascade, inducing the binding of heat shock element (HSE) with promoters of HSPs (Miller and Mittler, 2006; Li B. et al., 2018). Phytohormones, such as IAA, CKs, ABA, ET, GA, SA, BRs, and JA, contribute to the signal transduction pathways during heat stress (Eyidogan et al., 2012; Li N. et al., 2021). Several studies indicated calcineurin b-like protein-interacting protein kinase (CIPK) and named sucrose non-fermenting 1-related kinase (SnRK) family members as key players in pollen tube growth seed-set and abiotic stress by perceiving and mediating Ca^2 +^ signaling (Yang et al., 2008; Zhou et al., 2015). The Ca^2+^ signaling plays an essential role in the heat tolerance of seed-set in maize under field conditions. In this, calcium, as the critical secondary messenger converges signals transmitted from high temperature, membrane fluidity, calcium efflux, and ABA (among others), amplifies them through activation downstream of genes, such as *HSFA6b*, *ABF1*, *CYCD5;1*, MutS2, and HSPs during reproductive stage via the MAPK pathway (Figure 3 and Tables 1, 2), and eventually enhancing maize tolerance to heat stress for seed-set (Gao et al., 2019).

**TABLE 2:** Key genes related to heat stress mechanisms.

Genes	Gene description	Function	References
*Zm00001d044732* ABA	ABA-induced protein	Acts as a signaling hormone in plants against abiotic stress, but its function in energy homeostasis under heat stress	Cheikh and Jones, 1994
*Zm00001d045675* (*AS*)	Asparagine synthetase homolog 1	Elevated maximum daily temperature induces alternative splicing and the roles of SR (serine/arginine-rich) 45a	Li and Howell, 2021
*Zm00001d047847 (SR45a)*	Serine/arginine-rich splicing factor SR45a	Elevated maximum daily temperature induces alternative splicing and the roles of SR (serine/arginine-rich) 45a	Li and Howell, 2021
*GRMZM2G388045 GAMETE EXPRESSED 1 (GEX1)*	Encode GAMETE EXPRESSED 1 (GEX1)	Protective roles for reproductive stage under HS	Gao et al., 2019
*GRMZM2G377194 CYCD5;1*	Encode cyclin D5;1	Protective roles for reproductive stage under HS Increased seed set	Gao et al., 2019
*GRMZM2G406715*	Encodes a bZIP transcription factor		Gao et al., 2019
*GRMZM2G062914 (MPK14)*	Expresses a maize mitogen-activated protein kinase, MPK14.	Its Arabidopsis ortholog is *AtMPK1* can mediate and augment ABA signaling	Gao et al., 2019
*GRMZM2G059225 (ARF)*	Discolored-paralog3 putative ARF GTPase-activating domain protein with ankyrin repeat-containing protein	GTPase activator activity	Gao et al., 2019
*Zm00001d028408* (*HSP26)*	Heat shock protein 18 (Heat shock protein 26)	Early heat stress marker gene	Nieto-Sotelo et al., 1990; Abou-Deif et al., 2019
*Zm00001d006036 (ZmHSP70)*	Heat shock 70 kDa protein	Heat stress response Induced by heat in diurnal temperature cycles	Rashed et al., 2021
*Zm00001d003554 (ZmHSP22)*	22.0 kDa class IV heat shock protein	Heat stress response Induced by heat in diurnal temperature cycles	Rashed et al., 2021
*Zm00001d028557 (ZmHSP17.9)*	17.9 kDa class I heat shock protein	Heat stress response Induced by heat in diurnal temperature cycles	Rashed et al., 2021
*Zm00001d047542 (ZmHSP17.6)*	17.6 kDa class II heat shock protein	Heat stress response Induced by heat in diurnal temperature cycles	Rashed et al., 2021
*Hsp18.2*	Heat shock protein 18.2	Heat stress response	Borghi, 2010
*HSP90*	Heat shock protein 90	Induced by heat in diurnal temperature cycles	Marrs et al., 1993
*Zm00001d038806 (HSP101)*	Heat shock protein 101	Induced by heat in diurnal temperature cycles Play essential roles in both induced and basal thermotolerance and primary root growth	Nieto-Sotelo et al., 2002
*Zm00001d014090*	Mitochondrial heat shock protein 60	Induced by heat in diurnal temperature cycles	Prasad et al., 1990
*GRMZM2G409658*	Encodes a Calcineurin b-like protein-interacting protein kinase (CIPK)	Involved in the stress response process Function in signal transduction	Gao et al., 2019
*GRMZM2G116452*	Encodes Peroxidase superfamily protein	Involved in the stress response process	Gao et al., 2019
*GRMZM2G060349*	Encodes a DNA mismatch repair protein, MutS2	Upregulated by high temperature Involved in the stress response process	Gao et al., 2019
*GRMZM2G023081*	Encodes a cysteine-rich domain-containing protein	Involved in the stress response process	Gao et al., 2019
*GRMZM2G061515*	Auxin-responsive GH3 family protein expresses an indole-3-acetic acid-amido synthetase	Involved in the stress response process Function in signal transduction Involved in maintaining auxin homeostasis *in vivo* through catalyzing excess IAA conjugation to amino acids	Ludwig-Müller, 2011
*GRMZM2G377194*	Encodes a D-type cyclin, CYCD5;1	Upregulated by high temperature	Gao et al., 2019
*GRMZM2G026892*	Encodes a cysteine-rich protein (CRP)	Lose its stability under HS, and thus mean that it is unable to protect the process of seed-set	Gao et al., 2019
*GRMZM2G176605*	Encodes an ankyrin repeat domain-containing protein	Both pollen tube growth and germination are damaged due to the downregulation of an ankyrin repeat-containing protein	Huang et al., 2006
*ZmHSP17.0 and Zm00014a_022730 (ZmHSP17.8)*	Heat shock protein 17.2 and Heat shock protein, respectively	Form dodecamers at temperatures lower than heat shock (HS) Protect cellular proteins from aggregation during times of heat stress	Klein et al., 2014
*chloroplast sHSP26*	Small heat shock protein, chloroplastic	Involved in maize heat tolerance	Hu et al., 2015
*Zm00014a018076 ZmHSP16.9*	Class I heat shock protein 1	Expressed in root, leaf, and stem tissues under 40°C treatment, which HS and exogenous H_2_O_2_ upregulate	Sun et al., 2012
*Zm00001d028325*	brs1;brassinosteroid synthesis1	Confers thermotolerance	Dhaubhadel et al., 1999
*Zm00001d029149*	Zinc finger protein CONSTANS-LIKE 13	Heat response gene	Jagtap et al., 2020
*Zm00001d029892*	Metalloendoproteinase 1-MMP	Heat response gene	Jagtap et al., 2020
*Zm00001d033805*	Glutamate decarboxylase 1 (GAD 1)	Heat response gene Ca2+/calmodulin has been shown to bind GAD and stimulate its activity	Sachs et al., 1996; Jagtap et al., 2020
*Zm00001d002597*	Rho GTPase-activating protein 3	Heat response gene	Jagtap et al., 2020
*Zm00001d003643*	L-Ascorbate peroxidase S chloroplastic/mitochondrial	Heat response gene	Jagtap et al., 2020
*Zm00001d006036*	Heat shock 70 kDa protein 9 mitochondrial	Heat response gene	Jagtap et al., 2020
*Zm00001d041701*	Acyl carrier protein 2 chloroplastic	Heat response gene	Jagtap et al., 2020
*Zm00001d048592*	rca2; RUBISCO activase2: encodes the beta form of RUBISCO activase	Heat response gene	Jagtap et al., 2020
*Zm00001d051056*	*S*-adenosylmethionine decarboxylase proenzyme	Heat response gene	Jagtap et al., 2020
*Zm00001d017729*	Serine/threonine-protein kinase MHK	Heat response gene	Jagtap et al., 2020
*Zm00001d017992*	Metalloendoproteinase 1	Heat response gene	Jagtap et al., 2020
*Zm00001d037273*	Peptide methionine sulfoxide reductase msrB	Heat response gene	Jagtap et al., 2020
*Zm00001d037663*	NADH-ubiquinone oxidoreductase 10.5 kDa subunit	Heat response gene	Jagtap et al., 2020
*Zm00001d039188*	Putative leucine-rich repeat receptor-like protein kinase family protein	Heat response gene	Jagtap et al., 2020
*Zm00001d011760*	DNAJ heat shock N-terminal domain-containing protein	Heat response gene DNAJ proteins are co-chaperones of the Hsp70 machine, which play a critical role by stimulating Hsp70 ATPase activity, thereby stabilizing its interaction with client proteins	Pegoraro et al., 2011; Jagtap et al., 2020
*ZmNIP2-3*	Aquaporin NOD26-like membrane integral protein	Heat response gene Differentially phosphorylated under heat stress Encode aquaporins involved in silicon transport	Brusamarello-Santos et al., 2012; Jagtap et al., 2020
*Zm00001d045220*	Late embryogenesis abundant protein group 2	Heat response gene The LEA proteins are a family of hydrophilic proteins presumed to play a protective role during exposure to different abiotic stresses	Amara et al., 2013; Jagtap et al., 2020
*Zm00001d046363*	*S*-adenosyl-L-methionine-dependent methyltransferases superfamily protein	Heat response gene	Jagtap et al., 2020
*Zm00001d002262*	Uncharacterized LOC100502514	High grain yield QTL is related to heat stress	Frey et al., 2016
*Zm00001d005002*	Carbohydrate transporter/sugar porter/transporter	High grain yield QTL is related to heat stress	Frey et al., 2016
*Zm00001d004960*	Uncharacterized LOC100281571	High grain yield QTL is related to heat stress	Frey et al., 2016
*Zm00001d043407*	Uncharacterized LOC100282523	High grain yield QTL is related to heat stress	Frey et al., 2016
*Zm00001d013918*	Thylakoid lumenal 17.4 kDa protein chloroplastic	High grain yield QTL is related to heat stress	Frey et al., 2016
*Zm00001d047096*	Beta-expansin 1a	High grain yield QTL is related to heat stress	Frey et al., 2016

### Heat Stress-Mediated Transcriptional Regulation

Heat stress stimulates transcription of heat stress factors (HSFs) (Table 1) which subsequently results in overexpression of HSPs to mitigate the effect of heat stress (El-Sappah et al., 2012, 2017). However, only HSF or HSP overexpression has no significant role in heat tolerance, indicating that both gene families act synergistically (Wang et al., 2004).

Maize contains 25 HSFs, further divided into A, B, and C subclasses (Lin et al., 2011). Class A HSFs contribute to transcriptional activation, whereas the rest two classes have no specific role in transcriptional activation due to the absence of specific protein motifs (Reddy et al., 2014; Haider et al., 2021). A master transcription activator *HSFA1* stimulates immediate expression of different heat shock responsive transcription factors (TFs), including dehydration responsive element binding protein 2A (*DREB2A*), *HSFA2*, *HSFA7*, HSFBs, and multiprotein-bridging factor 1C (*MBF1C*) (Yan et al., 2020; Zhao J. et al., 2021). Additionally, heat stress stimulates the transactivation of *HSFA1* upon the interaction between *Hsp70* and *Hsp90* (Ohama et al., 2017).

*HSFA1* is comprised two alleles; *HSFA1a* and *HSFA1b* (El-Shershaby et al., 2019). *HSFA1* stimulates transcription of ERF/AP2 and *DREB2A* (Mizoi et al., 2012), *HSFA2* acts as a heat-inducible trans-activator of different genes (Chauhan et al., 2013), and *HSFA3* regulates the expression of *DREB2A* and *DREB2C* (Chen et al., 2010). *ZmHsf-6* belongs to class A1, *ZmHsf-1*, *ZmHsf-4*, *ZmHsf-5*, and *ZmHsf-17* belong to subclass A2, *ZmHsf-3*, *ZmHsf-11*, and *ZmHsf-25* belong to class B, all contributing key roles in heat tolerance in maize (Table 1; Lin et al., 2011; Zhang et al., 2020b; Jiang et al., 2021). The expression of *ZmHsf-6* was localized in pollens, and its expression was upregulated under heat stress (Jiang et al., 2021). Furthermore, *Hsp70-2* and *Hsp70-4* are downstream targets of *ZmHsf-6* and contribute significantly to abiotic stress response (Li H.-C. et al., 2015). The highest expression of *ZmHsf-1*, *ZmHsf-3*, and *ZmHsf-23* was observed in maize plants exposed to heat stress proving their significant role in maize during heat stress (Tables 1, 2; Lin et al., 2011).

In maize, ZmAP2/ERF is the most prominent TFs family comprised of 292 potential members, out of which 153 belong to the ERF subfamily (Zhou et al., 2012). Also, *ZmDREB2A* plays an essential role in heat tolerance and during drought tolerance in maize plants (Qin et al., 2007) when subjected to heat stress, *DREB2A* regulates transcription of *HSFA3* by stimulating coactivation complex comprised of NUCLEAR FACTOR Y, SUBUNIT A2 (NF-YA2), DNA POLYMERASE II SUBUNIT B3-1 (DPB3-1)/NF-YC10, and NF-YB3 (Schramm et al., 2008). Additionally, heat stress causes the over-accumulation of secondary heat stress-responsive ROS, with *HSFA4a* and *HSFA8* acting as sensors (Cimini et al., 2019; Xu et al., 2021). The maize genome contains 72 MYB TFs, with only 46 playing a key role in abiotic stress response (Du et al., 2012; Chen Y. et al., 2018). Maize plants exposed to abiotic stress factors including heat, salinity, drought, cold, and ABA resulted in overexpression of *ZmMYB-R* (Table 2; Liu et al., 2012; Kimotho et al., 2019).

### Protein Homeostasis Under Heat Stress

Heat stress interrupts the molecular mechanism of proper protein folding in the ER, which is toxic to ER (Howell, 2013; Fragkostefanakis et al., 2016). Unfolded-protein response (UPR) is an adaptive change in ER that avoids the toxic effect of misfolded proteins (Figure 3; Vitale and Boston, 2008); however, prolonged toxicity resulted in programmed cell death (Iurlaro and Muñoz-Pinedo, 2016). UPR also stimulates the signaling cascade to send an ER message to the nucleus to initiate the expression of toxicity-responsive genes (Neill et al., 2019). ER stress activates UPR *via* splicing of *ZmbZIP60* transcripts with the help of kinase (IRE1) and membrane-localized TFs, such as *ZmbZIP17* and a type II membrane protein *ZmbZIP28* (Figure 3; Nawkar et al., 2018; Pastor-Cantizano et al., 2020). Both signaling factors bind, producing heterodimers resulting from the upregulation of stress-responsive genes (Gayral et al., 2020). N-terminal domain of *bZIP28* transcription factor face toward cytosol, whereas C-terminal domain face toward the lumen of ER (Liu et al., 2007). From ER, *bZIP28* was first associated with Sar1 GTPase for packaging inside COPII vesicles and then exported to Golgi bodies for modifications by the Golgi site-1 and site-2 proteases (S1P and S2P) (Chung et al., 2018; Pastor-Cantizano et al., 2020). Under heat stress, the N-terminus of bZIP28 is cleaved by S2P, released inside the cytosol, and finally transported to the nucleus. Similarly, IRE1 activates the *bZIP60* transcription factor by splicing and transporting to the nucleus (Reimold et al., 2000; Huang et al., 2017).

The second abiotic stress signaling pathway initiated from ER is comprised of IRE1, a splicing protein, namely kinase/ribonuclease, which activates the *bZIP60* transcription factor *via* proteolysis (Kørner et al., 2015; Pastor-Cantizano et al., 2020). When maize seedlings are exposed to heat stress, the transcript of *ZmbZIP60* is activated by splicing and transferred to the nucleus to induce the expression of HSPs (Li Z. et al., 2018). Another ER-localized *ZmbZIP17* transcription factor is activated under heat and ABA stress and subsequently transported into the nucleus to transactivate HSPs with the help of UPR (Cacas, 2015). HSPs maintain cell metabolites stability under heat stress (Efeoǧlu, 2009). Major HSPs which play a key role during heat tolerance in maize are *ZmHSP16.9*, *ZmsHSP17*, *ZmsHSP17.8*, *ZmsHSP26*, *ZmHSP68*, *ZmHSP70*, *ZmHSP90*, and *ZmHSP101* (Tables 1, 2; Sun et al., 2012; Klein et al., 2014; Kumar et al., 2019; Zhao Y. et al., 2021). For example, when maize plants are exposed to heat stress at the reproductive stage, *ZmHSP101* is overexpressed in pollens to prevent their mortality, keep them viable and result in more grains (Gurley, 2000). Generally, transcriptome studies of four heat-tolerant and four heat-susceptible inbred lines, 607 heat-responsive genes, and 39 heat-tolerance genes were identified (Frey et al., 2015).

## Approaches for Improving Thermotolerance

### Agronomic Management

Several agronomic management practices, such as soil and nutrients management, crop rotation, plantation rate, timing, and irrigation, are beneficial in heat tolerance in maize (Sabagh et al., 2020). For example, early sowing of longer season varieties can overcome heat stress in spring maize (Liu et al., 2013). Similarly, nighttime subsurface drip irrigation reduces the root-zone causes in soil temperature, resulting in improved growth and yield in maize (Dong et al., 2016). Additionally, optimized irrigation enhances water use efficiency and aids heat tolerance (Tao and Zhang, 2010). Maize crops exposed to drought and heat stresses during vegetative growth are likely to have shortened reproductive growth stage, resulting in yield loss; however, they can be managed by maintaining soil moisture contents at 65% *via* drip irrigation (Yuan et al., 2004).

Heat stress negatively affects the absorption of adequate concentrations of minerals and nutrients required for normal metabolic and physiological processes (Fahad et al., 2017). For example, nitrogen (N) and magnesium (Mg) are structural parts of chlorophyll, phosphorus (P) is a structural part of nucleic acids (DNA and RNA), and potassium is required for osmotic regulation and activation of enzymes (Waraich et al., 2012; Meena et al., 2020). Additionally, nitrogen plays a key role in utilizing absorbed light, carbon assimilation, and heat tolerance (Meena et al., 2019). Thus, nutrient management can mitigate physiological disorders of maize plants exposed to heat stress, such as applying potassium (K), improving membranes’ stability, and maintaining turgor pressure in maize (Tao and Zhang, 2010). Specifically, nutrient management at the grain-filling stage contributes significantly to increased yield. Additionally, applying bioregulators, such as Putrescine and Thiourea, improved heat tolerance in maize seedlings (Yadav et al., 2017).

Exogenous application of plant growth regulators, such as ABA and CaCl_2_, play a key role in heat tolerance in maize by improving the capability of PSII and stopping the ROS, respectively (Gong et al., 1997; Tao and Zhang, 2010). Artificial application of auxin also improves abiotic stresses, including drought, salinity, waterlogging, heat and cold stress, UV irradiation, and heavy metals tolerance (Vardhini and Anjum, 2015). Similarly, the CK application helps mitigate the denaturation of proteins metabolites due to over-accumulation of ROS and improves the rate of photosynthesis in maize (Zulfiqar and Ashraf, 2021). Additionally, the application of SA and ABA ameliorate the effects of abiotic stress factors, improve seedling growth, mitigate ROS, stimulate the cell-signaling pathway via biosynthesis of NO, resulting in enhanced plant growth and crop yield (Meena et al., 2015). Exogenous application of GA improves the growth and development of plants via mitigating adverse effects of abiotic stresses (Yamaguchi, 2008). The BRs are a newly discovered group of plant hormones with promising potential in abiotic stress tolerance, ROS tolerance, and heat stress tolerance (Arif et al., 2020).

### Conventional Breeding

Availability of genetic variations in a population and relationship among traits is base for any successful plant breeding program (Lorenz et al., 2011; Aruah et al., 2012). The exact knowledge of genetic parameters, including population structure, heritability, and genotypic variance among the traits under selection pressure, helps develop efficient breeding lines (Farshadfar et al., 2013). In traditional breeding, selection procedures have been developed to identify and subsequently multiply maize verities with improved heat tolerance (Gong et al., 2015; Gedil and Menkir, 2019). Breeding heat-tolerant varieties is an effective strategy for improving heat tolerance in the spring maize grain-filling stage (Mishra et al., 2021). Many maize cultivars have been screened for canopy structure, flag leaf stomata, and rate of photosynthesis to obtain maximum yield and heat tolerance (Sah et al., 2020). The application of genetic markers accompanied by next-generation sequencing (NGS) has accelerated various development in breeding techniques (Le Nguyen et al., 2019).

### Quantitative Trait Locus and Marker-Assisted Breeding

Conventional breeding has significantly improved the selection of heat-tolerant crop varieties (Fu et al., 2012; Bai et al., 2018). During heat stress at the reproductive stage, quantitative trait locus (QTLs) play a major role in pollen production and preservation, receptivity and pollen tube development, proper grain-filling, and post-anthesis leaf senescence (Tiwari and Yadav, 2019). Therefore, the number and origin of QTLs are pivotal to mitigating heat stress (Sharma et al., 2017). Also, the number of QTLs and their roles studied in heat stress-exposed maize seedlings were six during pollen heat tolerance (Tiwari and Yadav, 2019), 11 at two different loci (HSI_*DY*_ and HSI_*DYA*_) during grain-filling located on chromosomes 2, 3, 5, and 9 (Frey et al., 2016) and six during heat susceptibility index (Van Inghelandt et al., 2019). Moreover, 6 and 5 QTLs have been detected associated with pollen tube growth and pollen germination, respectively, using a recombinant inbred population with 45 materials under abiotic stresses, including high temperature (Frova and Sari-Gorla, 1994; Frova et al., 1998). Therefore, these QTLs can be employed in conventional breeding to improve heat tolerance in maize cultivars (Frey et al., 2015). Previously explored maize QTLs can be assessed by exploring the following datasets; http://www.maizegdb.org and http://www.plantstress.com.

Quantitative trait locus are being widely employed in the introgression of favorable alleles in elite maize cultivars *via* backcrossing and confirmation in F1 (Frey et al., 2016; Cerrudo et al., 2018). Molecular markers including simple sequence repeats (SSR), single nucleotide polymorphisms (SNPs), random amplified polymorphic DNA (RAPD), and amplified fragment length polymorphism associated with heat tolerance are also employed in MAS (El-Sappah et al., 2019; Younis et al., 2020). The SNP and SSR are vast in identifying genotypes with maximum heat tolerance (Sabagh et al., 2020). Genome-wide association study (GWAS) is also a valuable tool in identifying novel QTLs responsible for heat tolerance to improve the genetic pool in maize breeding (Wen et al., 2014; Lafarge et al., 2017; Lin et al., 2020). GWAS is also helpful in revealing the linkage between SNPs and specific traits that confers heat tolerance at the flowering stage (Lafarge et al., 2017). GWAS was performed in sub-tropical maize, identifying significant SNPs and haplotype blocks associated with yield contributing traits that help select donor lines with favorable alleles for multiple characteristics, providing insights into heat stress tolerance genetics (Longmei et al., 2021; Seetharam et al., 2021).

### Genetic and Metabolic Engineering

Recently, several gene families have been identified and subsequently characterized in maize involved in heat stress response, such as heat shock protein-70 and heat shock factor (Casaretto et al., 2016; Jagtap et al., 2020; Jiang et al., 2021). Additionally, transcriptomic profiling of maize seedlings exposed to heat stress showed several differentially expressed genes employed in developing improved heat-tolerant maize varieties using robust genome editing techniques, such as RNAi and CRISPR/Cas9 (El-Sappah et al., 2021; Razzaq et al., 2021; Singh et al., 2021). Integration of robust genetic engineering techniques has accelerated conventional breeding of maize by reducing the time of variety development with the application of genetic markers in the early detection of desired traits in F1 (Ahmar et al., 2020). Furthermore, fast growth in NGS has enabled high throughput sequencing of desired traits which is cost-effective, time-saving, reproducible, impossible to achieve *via* conventional breeding (Kulski, 2016).

In maize, several genes have been identified to develop genetically modified (GMO) or transgenic verities with improved heat tolerance (Tiwari and Yadav, 2019; Malenica et al., 2021). For example, overexpression of *ZmVPP1* and *OsMYB55* resulted in increased heat and drought tolerance in maize (Casaretto et al., 2016; Wang et al., 2016). Furthermore, the HSFs gene family plays a pivotal role during heat stress (Haider et al., 2021). Up to 25 HSFs have been reported in several cereal crops, and their key role is confirmed in regulating Hsp genes (Guo et al., 2008). This discovery of identifying and characterizing HSFs and their role in regulating Hsp genes has provided a fundamental basis for the development of GM maize with the highest heat stress tolerance (Ahuja et al., 2010). Furthermore, the overexpression of chloroplast localized 6-phosphogluconate dehydrogenase (6PGDH) PGD3 displayed an over-accumulation of starch in maize endosperm under heat stress improved grain size and weight, whereas, *WPGD1* and *WPGD2* transgenes can increase the number of kernels to mitigate losses in high nighttime temperature conditions (Ribeiro et al., 2020). In the metabolic studies of Joshi et al. (2021), a total of 5,136 genes expression were affected in response to heat stress.

## Conclusion

Plant growth, development, and productivity are significantly affected by abiotic or biotic stresses because the plants, as sessile organisms, cannot move to favorable environments. Globally, high temperature has become a significant stressor that has accelerated the increase in the air temperature in the recent decades. Maize is a C4 crop species that belongs to the Poaceae family and is moderately sensitive to abiotic stresses, such as temperature. Maize plants are considered to be heat tolerant, but an extended duration of a temperature >35°C is deemed to be unfavorable for the development and growth of crops. In comparison, temperatures above 40°C, mainly during flowering and grain-filling season, will negatively affect the grain productivity of grain in these plants.

Heat stress may alter several physiological processes, namely membrane fluidity, net photosynthesis, respiration rate, hormone levels, osmolytes accumulation, and so on. High temperatures are related to several metabolic events at cellular and sub-cellular levels, leading to the production of ROS and oxidative stress. The anti-oxidative defense system is a prospective mechanism to protect them from ROS damage in plants. Finally, several agronomic management practices, such as the management of soil and nutrients, crop rotation, plantation rate, timing, and irrigation, are beneficial in developing heat tolerance in maize, along with advanced genomic tools. This review summarizes heat stress sensing, the induction of signaling cascade, symptoms, heat stress-related genes, molecular feature of maize response, and approaches to establishing heat-tolerant maize varieties.

## Future Perspectives

Environmental factors affecting maize growth and development include rainfall, light intensity, temperature (heat and cold), relative humidity, heavy metal stress, and wind speed. Drought and heat stress have severe implications for sustainable crop yield. Therefore, it is necessary to develop maize verities having maximum tolerance against drought and heat stress with breeding and genetic engineering. Although substantial efforts had been made to develop heat-tolerant maize verities *via* conventional breeding, it has limitations, such as being laborious, time-consuming, and the possibility of only intra-species gene transfer. However, modern genetic approaches, such as GWAS and genotyping by sequencing, have facilitated inter-species gene transfer to develop maize verities with the highest heat tolerance. Additionally, the complementation of conventional breeding with the development of modern and robust genetic engineering techniques, such as RNAi, CRISPR/Cas9, and TILLING, has accelerated the process of variety development.

## Author Contributions

AE-S, KE-T, JL, SAR, RRM, and MA: conceptualization. AE-S: writing original draft and collecting the data. AE-S and ASE: draw the figures. AE-S, KY, SHW, MB, QH, ZAD, MMAE, MK, RRM, JL, and KE-T: review, and editing of the manuscript. AE-S, KE-T, and MA: writing final copy. All authors contributed to the article and approved the submitted version.

## Conflict of Interest

The authors declare that the research was conducted in the absence of any commercial or financial relationships that could be construed as a potential conflict of interest.

## Publisher’s Note

All claims expressed in this article are solely those of the authors and do not necessarily represent those of their affiliated organizations, or those of the publisher, the editors and the reviewers. Any product that may be evaluated in this article, or claim that may be made by its manufacturer, is not guaranteed or endorsed by the publisher.

## References

[B1] Abou-Deif M. H. Rashed M. A.-S. Khalil K. M. Mahmoud F. E.-S. (2019). Proteomic analysis of heat shock proteins in maize (*Zea mays* L.). *Bull. Natl. Res. Cent.* 43:199.

[B2] Ahmar S. Gill R. A. Jung K.-H. Faheem A. Qasim M. U. Mubeen M. (2020). Conventional and molecular techniques from simple breeding to speed breeding in crop plants: recent advances and future outlook. *Int. J. Mol. Sci.* 21:2590. 10.3390/ijms21072590 32276445 PMC7177917

[B3] Ahmed I. Ur Rahman M. H. Ahmed S. Hussain J. Ullah A. Judge J. (2018). Assessing the impact of climate variability on maize using simulation modeling under semi-arid environment of Punjab, Pakistan. *Environ. Sci. Pollut. Res.* 25 28413–28430. 10.1007/s11356-018-2884-3 30083905

[B4] Ahuja I. De Vos R. C. Bones A. M. Hall R. D. (2010). Plant molecular stress responses face climate change. *Trends Plant Sci.* 15 664–674. 10.1016/j.tplants.2010.08.002 20846898

[B5] Alam M. R. Nakasathien S. Molla M. S. H. Islam M. A. Maniruzzaman M. Ali M. A. (2021). Kernel water relations and kernel filling traits in maize (*Zea mays* L.) are influenced by water-deficit condition in a tropical environment. *Front. Plant Sci.* 12:717178. 10.3389/fpls.2021.717178 34712250 PMC8546300

[B6] Amara I. Capellades M. Ludevid M. D. Pagès M. Goday A. (2013). Enhanced water stress tolerance of transgenic maize plants over-expressing LEA Rab28 gene. *J. Plant Physiol.* 170 864–873. 10.1016/j.jplph.2013.01.004 23384757

[B7] Arif Y. Singh P. Siddiqui H. Bajguz A. Hayat S. (2020). Salinity induced physiological and biochemical changes in plants: an omic approach towards salt stress tolerance. *Plant Physiol. Biochem.* 156 64–77. 10.1016/j.plaphy.2020.08.042 32906023

[B8] Aruah B. C. Uguru M. I. Oyiga B. C. (2012). Genetic variability and inter-relationship among some Nigerian pumpkin accessions (*Cucurbita* spp.). *Int. J. Plant Breed.* 6 34–41.

[B9] Atkinson N. J. Lilley C. J. Urwin P. E. (2013). Identification of genes involved in the response of Arabidopsis to simultaneous biotic and abiotic stresses. *Plant Physiol.* 162 2028–2041. 10.1104/pp.113.222372 23800991 PMC3729780

[B10] Bai S. Yu H. Wang B. Li J. (2018). Retrospective and perspective of rice breeding in China. *J. Genet. Genomics* 45 603–612. 10.1016/j.jgg.2018.10.002 30449538

[B11] Banerjee A. Roychoudhury A. (2018). “Abiotic stress, generation of reactive oxygen species, and their consequences: an overview,” in *Reactive Oxygen Species in Plants: Boon or Bane? Revisiting the Role of ROS*, eds Singh V. P. Singh S. Tripathi D. Mohan Prasad S. Chauhan D. K. (Hoboken, NJ: John Wiley & Sons Ltd), 23–50. 10.1002/9781119324928.ch2

[B12] Bartels D. Sunkar R. (2005). Drought and salt tolerance in plants. *CRC Crit. Rev. Plant Sci.* 24 1446–1452.

[B13] Bassani M. Neumann P. M. Gepstein S. (2004). Differential expression profiles of growth-related genes in the elongation zone of maize primary roots. *Plant Mol. Biol.* 56 367–380. 10.1007/s11103-004-3474-y 15604750

[B14] Baus D. (2017). *Overpopulation and the Impact on the Environment*. Master thesis. New York, NY: City University of New York.

[B15] Bechtold U. Penfold C. A. Jenkins D. J. Legaie R. Moore J. D. Lawson T. (2016). Time-series transcriptomics reveals that *AGAMOUS-LIKE22* affects primary metabolism and developmental processes in drought-stressed Arabidopsis. *Plant Cell* 28 345–366. 10.1105/tpc.15.00910 26842464 PMC4790877

[B16] Bedard K. Lardy B. Krause K. H. (2007). NOX family NADPH oxidases: not just in mammals. *Biochimie* 89 1107–1112. 10.1016/j.biochi.2007.01.012 17400358

[B17] Borghi L. (2010). Inducible gene expression systems for plants. *Plant Dev. Biol.* 655 65–75. 10.1007/978-1-60761-765-5_5 20734254

[B18] Bourgine B. Guihur A. (2021). Heat shock signaling in land plants: from plasma membrane sensing to the transcription of small heat shock proteins. *Front. Plant Sci.* 12:710801. 10.3389/fpls.2021.710801 34434209 PMC8381196

[B19] Bruns H. A. (2003). Controlling aflatoxin and fumonisin in maize by crop management. *J. Toxicol. Toxin Rev.* 22 153–173. 10.1081/txr-120024090

[B20] Brusamarello-Santos L. Pacheco F. Aljanabi S. Monteiro R. Cruz L. Baura V. (2012). Differential gene expression of rice roots inoculated with the diazotroph *Herbaspirillum seropedicae*. *Plant Soil* 356 113–125. 10.1007/s11104-011-1044-z

[B21] Cacas J.-L. (2015). “Out for a walk along the secretory pathway during programmed cell death,” in *Plant Programmed Cell Death*, eds Gunawardena A. H. McCabe P. F. (Powell, WY: Springer), 123–161. 10.1007/978-3-319-21033-9_6

[B22] Caine R. S. Yin X. Sloan J. Harrison E. L. Mohammed U. Fulton T. (2019). Rice with reduced stomatal density conserves water and has improved drought tolerance under future climate conditions. *New Phytol.* 221 371–384. 10.1111/nph.15344 30043395 PMC6492113

[B23] Casaretto J. A. El-Kereamy A. Zeng B. Stiegelmeyer S. M. Chen X. Bi Y.-M. (2016). Expression of OsMYB55 in maize activates stress-responsive genes and enhances heat and drought tolerance. *BMC Genomics* 17:312. 10.1186/s12864-016-2659-5 27129581 PMC4850646

[B24] Catalá A. (2009). Lipid peroxidation of membrane phospholipids generates hydroxy-alkenals and oxidized phospholipids active in physiological and/or pathological conditions. *Chem. Phys. Lipids* 157 1–11. 10.1016/j.chemphyslip.2008.09.004 18977338

[B25] Cerrudo D. Cao S. Yuan Y. Martinez C. Suarez E. A. Babu R. (2018). Genomic selection outperforms marker assisted selection for grain yield and physiological traits in a maize doubled haploid population across water treatments. *Front. Plant Sci.* 9:366. 10.3389/fpls.2018.00366 29616072 PMC5869257

[B26] Chao L. Zou J.-X. Peng S. Peng Y. Yuan S.-F. Xia W. (2016). causes and impacts for heat stress in spring maize during grain filling in the North China plain â a review. *J. Integr. Agric.* 15 2677–2687. 10.1016/s2095-3119(16)61409-0

[B27] Chapman J. M. Muhlemann J. K. Gayomba S. R. Muday G. K. (2019). RBOH-dependent ROS synthesis and ROS scavenging by plant specialized metabolites to modulate plant development and stress responses. *Chem. Res. Toxicol.* 32 370–396. 10.1021/acs.chemrestox.9b00028 30781949 PMC6857786

[B28] Chauhan H. Khurana N. Agarwal P. Khurana J. P. Khurana P. (2013). A seed preferential heat shock transcription factor from wheat provides abiotic stress tolerance and yield enhancement in transgenic *Arabidopsis* under heat stress environment. *PLoS One* 8:e79577. 10.1371/journal.pone.0079577 24265778 PMC3827158

[B29] Cheikh N. Jones R. J. (1994). Disruption of maize kernel growth and development by heat stress (role of cytokinin/abscisic acid balance). *Plant Physiol.* 106 45–51. 10.1104/pp.106.1.45 12232301 PMC159497

[B30] Chen H. Hwang J. E. Lim C. J. Kim D. Y. Lee S. Y. Lim C. O. (2010). *Arabidopsis* DREB2C functions as a transcriptional activator of HsfA3 during the heat stress response. *Biochem. Biophys. Res. Commun.* 401 238–244. 10.1016/j.bbrc.2010.09.038 20849812

[B31] Chen K. Ali S. Chen Y. Sohail A. Jan A. Fahad S. (2018). Effect of ridge-covering mulching materials on hormonal changes, antioxidative enzyme activities and production of maize in semi-arid regions of China. *Agric. Water Manag.* 204 281–291. 10.1016/j.agwat.2018.03.023

[B32] Chen Y. Cao Y. Wang L. Li L. Yang J. Zou M. (2018). Identification of MYB transcription factor genes and their expression during abiotic stresses in maize. *Biol. Plant.* 62 222–230. 10.1007/s10535-017-0756-1

[B33] Chung K. P. Zeng Y. Li Y. Ji C. Xia Y. Jiang L. (2018). Signal motif-dependent ER export of the Qc-SNARE BET12 interacts with MEMB12 and affects PR1 trafficking in *Arabidopsis*. *J. Cell Sci.* 131:jcs202838. 10.1242/jcs.202838 28546447

[B34] Cimini S. Gualtieri C. Macovei A. Balestrazzi A. De Gara L. Locato V. (2019). Redox balance-DDR-miRNA triangle: relevance in genome stability and stress responses in plants. *Front. Plant Sci.* 10:989. 10.3389/fpls.2019.00989 31428113 PMC6688120

[B35] Colasanti J. Muszynski M. (2009). “The maize floral transition,” in *Handbook of Maize: its Biology*, Vol. 1 eds Hake S. C. Bennetzen J. L. (New York, NY: Springer Science), 41–55. 10.1111/nph.16772

[B36] Crafts-Brandner S. J. Salvucci M. E. (2002). Sensitivity of photosynthesis in a C4 plant, maize, to heat stress. *Plant Physiol.* 129 1773–1780. 10.1104/pp.002170 12177490 PMC166765

[B37] Czarnocka W. Karpińskiski S. (2018). Friend or foe? Reactive oxygen species production, scavenging and signaling in plant response to environmental stresses. *Free Radic. Biol. Med.* 122 4–20. 10.1016/j.freeradbiomed.2018.01.011 29331649

[B38] Dai Z. Ku M. S. Edwards G. E. (1993). C4 photosynthesis (the CO2-concentrating mechanism and photorespiration). *Plant Physiol.* 103 83–90. 10.1104/pp.103.1.83 12231916 PMC158949

[B39] Dar Z. A. Dar S. A. Khan J. A. Lone A. A. Langyan S. Lone B. A. (2021). Identification for surrogate drought tolerance in maize inbred lines utilizing high-throughput phenomics approach. *PLoS One* 16:e0254318. 10.1371/journal.pone.0254318 34314420 PMC8315520

[B40] Daryanto S. Wang L. Jacinthe P.-A. (2016). Global synthesis of drought effects on maize and wheat production. *PLoS One* 11:e0156362. 10.1371/journal.pone.0156362 27223810 PMC4880198

[B41] Dawood M. Moursi Y. Amro A. Baenziger P. Sallam A. (2020). Investigation of heat-induced changes in the grain yield and grains metabolites, with molecular insights on the candidate genes in barley. *Agronomy* 10:1730. 10.3390/agronomy10111730

[B42] Dawson T. P. Perryman A. H. Osborne T. M. (2016). Modelling impacts of climate change on global food security. *Clim. Change* 134 429–440. 10.1007/s10584-014-1277-y

[B43] Demidchik V. (2015). Mechanisms of oxidative stress in plants: from classical chemistry to cell biology. *Environ. Exp. Bot.* 109 212–228. 10.1016/j.envexpbot.2014.06.021

[B44] Dhaubhadel S. Chaudhary S. Dobinson K. F. Krishna P. (1999). Treatment with 24-epibrassinolide, a brassinosteroid, increases the basic thermotolerance of *Brassica napus* and tomato seedlings. *Plant Mol. Biol.* 40 333–342. 10.1023/a:1006283015582 10412911

[B45] Dias M. Brüggemann W. (2010). Limitations of photosynthesis in *Phaseolus vulgaris* under drought stress: gas exchange, chlorophyll fluorescence and Calvin cycle enzymes. *Photosynthetica* 48 96–102. 10.1007/s11099-010-0013-8

[B46] Djanaguiraman M. Prasad P. V. Seppanen M. (2010). Selenium protects sorghum leaves from oxidative damage under high temperature stress by enhancing antioxidant defense system. *Plant Physiol. Biochem.* 48 999–1007. 10.1016/j.plaphy.2010.09.009 20951054

[B47] Dolatabadian A. Sanavy S. A. M. M. Asilan K. S. (2010). Effect of ascorbic acid foliar application on yield, yield component and several morphological traits of grain corn under water deficit stress conditions. *Not. Sci. Biol.* 2 45–50. 10.15835/nsb234717

[B48] Dong X. Xu W. Zhang Y. Leskovar D. I. (2016). Effect of irrigation timing on root zone soil temperature, root growth and grain yield and chemical composition in corn. *Agronomy* 6:34. 10.3390/agronomy6020034

[B49] Dordas C. (2009). Dry matter, nitrogen and phosphorus accumulation, partitioning and remobilization as affected by N and P fertilization and source–sink relations. *Eur. J. Agron.* 30 129–139. 10.1016/j.eja.2008.09.001

[B50] Du H. Feng B.-R. Yang S.-S. Huang Y.-B. Tang Y.-X. (2012). The R2R3-MYB transcription factor gene family in maize. *PLoS One* 7:e37463. 10.1371/journal.pone.0037463 22719841 PMC3370817

[B51] Duszyn M. świeżawska B. Szmidt-Jaworska A. Jaworski K. (2019). Cyclic nucleotide gated channels (CNGCs) in plant signalling—current knowledge and perspectives. *J. Plant Physiol.* 241:153035. 10.1016/j.jplph.2019.153035 31491601

[B52] Edreira J. I. R. Mayer L. I. Otegui M. E. (2014). Heat stress in temperate and tropical maize hybrids: kernel growth, water relations and assimilate availability for grain filling. *Field Crops Res.* 166 162–172. 10.1016/j.fcr.2014.06.018

[B53] Efeoǧlu B. (2009). Heat shock proteins and heat shock response in plants. *Gazi Univ. J. Sci.* 22 67–75.

[B54] Elferjani R. Soolanayakanahally R. (2018). Canola responses to drought, heat, and combined stress: shared and specific effects on carbon assimilation, seed yield, and oil composition. *Front. Plant Sci.* 9:1224. 10.3389/fpls.2018.01224 30214451 PMC6125602

[B55] El-Sappah A. Shawky A. Sayed-Ahmad M. Youssef M. (2012). Nile tilapia as bio indicator to estimate the contamination of water using SDS-PAGE and RAPDPCR techniques. *Egypt. J. Genet. Cytol.* 41 209–227. 10.21608/ejgc.2012.10536

[B56] El-Sappah A. H. Mm H. I El-Awady H. Yan S. Qi S. Liu J. (2019). Tomato natural resistance genes in controlling the root-knot nematode. *Genes* 10:925. 10.3390/genes10110925 31739481 PMC6896013

[B57] El-Sappah A. H. Rather S. A. (eds). (2022). “Genomics approaches to study abiotic stress tolerance in plants,” in *Plant Abiotic Stress Physiology*, Vol. 2 (Burlington: Apple Academic Press), 25. 10.1201/9781003180579-2

[B58] El-Sappah A. H. Shawky A. Sayed-Ahmad M. S. Youssef M. (2017). Estimation of heat shock protein 70 (hsp 70) gene expression in nile tilapia (*Oreochromis niloticus*) using quantitative real-time PCR. *Zagazig J. Agric. Res.* 44 1003–1015. 10.21608/zjar.2017.52300

[B59] El-Sappah A. H. Yan K. Huang Q. Islam M. M. Li Q. Wang Y. (2021). Comprehensive mechanism of gene silencing and its role in plant growth and development. *Front. Plant Sci.* 12:705249. 10.3389/fpls.2021.705249 34589097 PMC8475493

[B60] El-Sharkawy M. A. (2007). Physiological characteristics of cassava tolerance to prolonged drought in the tropics: implications for breeding cultivars adapted to seasonally dry and semiarid environments. *Braz. J. Plant Physiol.* 19 257–286. 10.1590/s1677-04202007000400003

[B61] El-Shershaby A. Ullrich S. Simm S. Scharf K.-D. Schleiff E. Fragkostefanakis S. (2019). Functional diversification of tomato HsfA1 factors is based on DNA binding domain properties. *Gene* 714:143985. 10.1016/j.gene.2019.143985 31330236

[B62] Erb M. Kliebenstein D. J. (2020). Plant secondary metabolites as defenses, regulators, and primary metabolites: the blurred functional trichotomy. *Plant Physiol.* 184 39–52. 10.1104/pp.20.00433 32636341 PMC7479915

[B63] Essemine J. Govindachary S. Ammar S. Bouzid S. Carpentier R. (2012). Enhanced sensitivity of the photosynthetic apparatus to heat stress in digalactosyl-diacylglycerol deficient Arabidopsis. *Environ. Exp. Bot.* 80 16–26. 10.1016/j.envexpbot.2011.12.022

[B64] Eyidogan F. Oz M. Yucel M. Oktem H. (2012). “Signal transduction of phytohormones under abiotic stresses,” in *Phytohormones and Abiotic Stress Tolerance in Plants*, eds Khan N. A. Nazar R. Iqbal N. Anjum N. A. (Berlin: Springer), 1–48. 10.1007/978-3-642-25829-9_1

[B65] Fahad S. Bajwa A. A. Nazir U. Anjum S. A. Farooq A. Zohaib A. (2017). Crop production under drought and heat stress: plant responses and management options. *Front. Plant Sci.* 8:1147. 10.3389/fpls.2017.01147 28706531 PMC5489704

[B66] Faostat F. (2017). Available Online at: http://www.fao.org/faostat/en/#data.QC [accessed January 2018].

[B67] Faostat F. (2019). *Food and Agriculture Organization of the United Nations-Statistic Division.* Available Online at: https://www.fao.org/faostat/en/#data.QC

[B68] Farshadfar E. Romena H. Safari H. (2013). Evaluation of variability and genetic parameters in agro-physiological traits of wheat under rain-fed condition. *Int. J. Agric. Crop Sci.* 5 1015–1021.

[B69] Finka A. Goloubinoff P. (2014). The CNGCb and CNGCd genes from *Physcomitrella patens* moss encode for thermosensory calcium channels responding to fluidity changes in the plasma membrane. *Cell Stress Chaperones* 19 83–90. 10.1007/s12192-013-0436-9 23666745 PMC3857430

[B70] Foyer C. H. Shigeoka S. (2011). Understanding oxidative stress and antioxidant functions to enhance photosynthesis. *Plant Physiol.* 155 93–100. 10.1104/pp.110.166181 21045124 PMC3075779

[B71] Fragkostefanakis S. Mesihovic A. Hu Y. Schleiff E. (2016). Unfolded protein response in pollen development and heat stress tolerance. *Plant Reprod.* 29 81–91. 10.1007/s00497-016-0276-8 27022919

[B72] Frederickson Matika D. E. Loake G. J. (2014). Redox regulation in plant immune function. *Antioxid. Redox Signal.* 21 1373–1388. 10.1089/ars.2013.5679 24206122 PMC4158969

[B73] Frey F. P. Presterl T. Lecoq P. Orlik A. Stich B. (2016). First steps to understand heat tolerance of temperate maize at adult stage: identification of QTL across multiple environments with connected segregating populations. *Theor. Appl. Genet.* 129 945–961. 10.1007/s00122-016-2674-6 26886101 PMC4835532

[B74] Frey F. P. Urbany C. Hüttel B. Reinhardt R. Stich B. (2015). Genome-wide expression profiling and phenotypic evaluation of European maize inbreds at seedling stage in response to heat stress. *BMC Genomics* 16:123. 10.1186/s12864-015-1282-1 25766122 PMC4347969

[B75] Frova C. Caffulli A. Pallavera E. (1998). Mapping quantitative trait loci for tolerance to abiotic stresses in maize. *J. Exp. Zool.* 282 164–170. 10.1002/(sici)1097-010x(199809/10)282:1/2<164::aid-jez18>3.0.co;2-u

[B76] Frova C. Sari-Gorla M. (1994). Quantitative trait loci (QTLs) for pollen thermotolerance detected in maize. *Mol. Gen. Genet.* 245 424–430. 10.1007/BF00302254 7808391

[B77] Fu J. Momcilovic I. Prasad P. Josipovic S. Ludwig E. (2012). “Molecular bases and improvement of heat tolerance in crop plants,” in *Heat Stress: Causes, Prevention and Treatments*, eds Josipovic S. Ludwig E. (Hauppauge, NY: Nova Science Publishers), 185–214.

[B78] Gao F. Han X. Wu J. Zheng S. Shang Z. Sun D. (2012). A heat-activated calcium-permeable channel–Arabidopsis cyclic nucleotide-gated ion channel 6–is involved in heat shock responses. *Plant J.* 70 1056–1069. 10.1111/j.1365-313X.2012.04969.x 22372427

[B79] Gao H. Brandizzi F. Benning C. Larkin R. M. (2008). A membrane-tethered transcription factor defines a branch of the heat stress response in *Arabidopsis thaliana*. *Proc. Natl. Acad. Sci. U.S.A.* 105 16398–16403. 10.1073/pnas.0808463105 18849477 PMC2571009

[B80] Gao J. Wang S. Zhou Z. Wang S. Dong C. Mu C. (2019). Linkage mapping and genome-wide association reveal candidate genes conferring thermotolerance of seed-set in maize. *J. Exp. Bot.* 70 4849–4864. 10.1093/jxb/erz171 30972421

[B81] Gao X. Cox K. L. Jr. He P. (2014). Functions of calcium-dependent protein kinases in plant innate immunity. *Plants* 3 160–176. 10.3390/plants3010160 27135498 PMC4844305

[B82] Gardeström P. Igamberdiev A. U. (2016). The origin of cytosolic ATP in photosynthetic cells. *Physiol. Plant.* 157 367–379. 10.1111/ppl.12455 27087668

[B83] Gayral M. Arias Gaguancela O. Vasquez E. Herath V. Flores F. J. Dickman M. B. (2020). Multiple ER-to-nucleus stress signaling pathways are activated during *Plantago asiatica* mosaic virus and *Turnip mosaic* virus infection in *Arabidopsis thaliana*. *Plant J.* 103 1233–1245. 10.1111/tpj.14798 32390256

[B84] Gedil M. Menkir A. (2019). An integrated molecular and conventional breeding scheme for enhancing genetic gain in maize in Africa. *Front. Plant Sci.* 10:1430. 10.3389/fpls.2019.01430 31781144 PMC6851238

[B85] Gong F. Wu X. Zhang H. Chen Y. Wang W. (2015). Making better maize plants for sustainable grain production in a changing climate. *Front. Plant Sci.* 6:835. 10.3389/fpls.2015.00835 26500671 PMC4593952

[B86] Gong M. Li Y.-J. Dai X. Tian M. Li Z.-G. (1997). Involvement of calcium and calmodulin in the acquisition of heat-shock induced thermotolerance in maize seedlings. *J. Plant Physiol.* 150 615–621. 10.1016/s0176-1617(97)80328-8

[B87] Gourdji S. M. Sibley A. M. Lobell D. B. (2013). Global crop exposure to critical high temperatures in the reproductive period: historical trends and future projections. *Environ. Res. Lett.* 8:024041. 10.1088/1748-9326/8/2/024041

[B88] Guo H. Li S. Kang S. Du T. Tong L. Ding R. (2019). Annual ecosystem respiration of maize was primarily driven by crop growth and soil water conditions. *Agric. Ecosyst. Environ.* 272 254–265. 10.1016/j.agee.2018.11.026

[B89] Guo J. Wu J. Ji Q. Wang C. Luo L. Yuan Y. (2008). Genome-wide analysis of heat shock transcription factor families in rice and *Arabidopsis*. *J. Genet. Genomics* 35 105–118. 10.1016/S1673-8527(08)60016-8 18407058

[B90] Gurley W. B. (2000). HSP101: a key component for the acquisition of thermotolerance in plants. *Plant Cell* 12 457–460. 10.1105/tpc.12.4.457 10760235 PMC526003

[B91] Haider S. Rehman S. Ahmad Y. Raza A. Tabassum J. Javed T. (2021). In silico characterization and expression profiles of heat shock transcription factors (HSFs) in maize (*Zea mays* L.). *Agronomy* 11:2335. 10.3390/agronomy11112335

[B92] Hao L. Qiao X. (2018). Genome-wide identification and analysis of the CNGC gene family in maize. *PeerJ* 6:e5816. 10.7717/peerj.5816 30356996 PMC6195792

[B93] Hasanuzzaman M. Bhuyan M. Zulfiqar F. Raza A. Mohsin S. M. Mahmud J. A. (2020). Reactive oxygen species and antioxidant defense in plants under abiotic stress: revisiting the crucial role of a universal defense regulator. *Antioxidants* 9:681. 10.3390/antiox9080681 32751256 PMC7465626

[B94] Hashimoto K. Kudla J. (2011). Calcium decoding mechanisms in plants. *Biochimie* 93 2054–2059. 10.1016/j.biochi.2011.05.019 21658427

[B95] Haswell E. S. Verslues P. E. (2015). The ongoing search for the molecular basis of plant osmosensing. *J. Gen. Physiol.* 145 389–394. 10.1085/jgp.201411295 25870206 PMC4411250

[B96] Hayes S. Schachtschabel J. Mishkind M. Munnik T. Arisz S. A. (2021). Hot topic: thermosensing in plants. *Plant Cell Environ.* 44 2018–2033. 10.1111/pce.13979 33314270 PMC8358962

[B97] He F. Gao C. Guo G. Liu J. Gao Y. Pan R. (2019). Maize annexin genes ZmANN33 and ZmANN35 encode proteins that function in cell membrane recovery during seed germination. *J. Exp. Bot.* 70 1183–1195. 10.1093/jxb/ery452 30649398 PMC6382337

[B98] He Y.-Y. Häder D.-P. (2002). UV-B-induced formation of reactive oxygen species and oxidative damage of the cyanobacterium *Anabaena* sp.: protective effects of ascorbic acid and N-acetyl-L-cysteine. *J. Photochem. Photobiol. B Biol.* 66 115–124. 10.1016/s1011-1344(02)00231-2 11897511

[B99] Hoegh-Guldberg O. Jacob D. Taylor M. Bolaños T. G. Bindi M. Brown S. (2019). The human imperative of stabilizing global climate change at 1.5°C. *Science* 365:eaaw6974. 10.1126/science.aaw6974 31604209

[B100] Horváth I. Glatz A. Nakamoto H. Mishkind M. L. Munnik T. Saidi Y. (2012). Heat shock response in photosynthetic organisms: membrane and lipid connections. *Prog. Lipid Res.* 51 208–220. 10.1016/j.plipres.2012.02.002 22484828

[B101] Hossain M. A. Bhattacharjee S. Armin S. M. Qian P. Xin W. Li H. Y. (2015). Hydrogen peroxide priming modulates abiotic oxidative stress tolerance: insights from ROS detoxification and scavenging. *Front. Plant Sci.* 6:420. 10.3389/fpls.2015.00420 26136756 PMC4468828

[B102] Houghton J. T. Ding Y. Griggs D. J. Noguer M. Van Der Linden P. J. Dai X. (2001). *Climate Change 2001: the Scientific Basis: Contribution of Working Group I to the Third Assessment Report of the Intergovernmental Panel on Climate Change.* Cambridge: Cambridge university press.

[B103] Howell S. H. (2013). Endoplasmic reticulum stress responses in plants. *Annu. Rev. Plant Biol.* 64 477–499. 10.1146/annurev-arplant-050312-120053 23330794

[B104] Hu X. Huang Y. Sun W. Yu L. (2017). Shifts in cultivar and planting date have regulated rice growth duration under climate warming in China since the early 1980s. *Agric. For. Meteorol.* 247 34–41. 10.1016/j.agrformet.2017.07.014

[B105] Hu X. Jiang M. Zhang J. Zhang A. Lin F. Tan M. (2007). Calcium/calmodulin is required for abscisic acid-induced antioxidant defense and functions both upstream and downstream of H_2_O_2_ production in leaves of maize plants. *New Phytol.* 173 27–38. 10.1111/j.1469-8137.2006.01888.x 17176391

[B106] Hu X. Yang Y. Gong F. Zhang D. Zhang L. Wu L. (2015). Protein sHSP26 improves chloroplast performance under heat stress by interacting with specific chloroplast proteins in maize (*Zea mays*). *J. Proteom.* 115 81–92. 10.1016/j.jprot.2014.12.009 25540934

[B107] Huang H.-W. Zeng X. Rhim T. Ron D. Ryoo H. D. (2017). The requirement of IRE1 and XBP1 in resolving physiological stress during *Drosophila* development. *J. Cell Sci.* 130 3040–3049. 10.1242/jcs.203612 28775151 PMC5612175

[B108] Huang J. Chen F. Del Casino C. Autino A. Shen M. Yuan S. (2006). An ankyrin repeat-containing protein, characterized as a ubiquitin ligase, is closely associated with membrane-enclosed organelles and required for pollen germination and pollen tube growth in lily. *Plant Physiol.* 140 1374–1383. 10.1104/pp.105.074922 16461387 PMC1435812

[B109] Huang Y.-C. Niu C.-Y. Yang C.-R. Jinn T.-L. (2016). The heat stress factor HSFA6b connects ABA signaling and ABA-mediated heat responses. *Plant Physiol.* 172 1182–1199. 10.1104/pp.16.00860 27493213 PMC5047099

[B110] Ihsan M. Z. Daur I. Alghabari F. Alzamanan S. Rizwan S. Ahmad M. (2019). Heat stress and plant development: role of sulphur metabolites and management strategies. *Acta Agric. Scand. B Soil Plant Sci.* 69 332–342. 10.1080/09064710.2019.1569715

[B111] Iurlaro R. Muñoz-Pinedo C. (2016). Cell death induced by endoplasmic reticulum stress. *FEBS J.* 283 2640–2652. 26587781 10.1111/febs.13598

[B112] Jagadish S. Bahuguna R. N. Djanaguiraman M. Gamuyao R. Prasad P. Craufurd P. Q. (2016). Implications of high temperature and elevated CO2 on flowering time in plants. *Front. Plant Sci.* 7:913. 10.3389/fpls.2016.00913 27446143 PMC4921480

[B113] Jagtap A. B. Vikal Y. Johal G. S. (2020). Genome-wide development and validation of cost-effective KASP marker assays for genetic dissection of heat stress tolerance in maize. *Int. J. Mol. Sci.* 21:7386. 10.3390/ijms21197386 33036291 PMC7582619

[B114] Jammes F. Hu H. C. Villiers F. Bouten R. Kwak J. M. (2011). Calcium-permeable channels in plant cells. *FEBS J.* 278 4262–4276. 10.1111/j.1742-4658.2011.08369.x 21955583

[B115] Jha A. K. Chakraborty S. Kumari K. Bauddh K. (2020). “Ecological consequences of genetically modified crops on soil biodiversity,” in *Ecological and Practical Applications for Sustainable Agriculture*, eds Bauddh K. Kumar S. Singh R. Korstad J. (Singapore: Springer), 89–106. 10.1007/978-981-15-3372-3_5

[B116] Jia Z. Lian Y. Zhu Y. He J. Cao Z. Wang G. (2009). Cloning and characterization of a putative transcription factor induced by abiotic stress in *Zea mays*. *Afr. J. Biotechnol.* 8 6764–6771.

[B117] Jiang L. Hu W. Qian Y. Ren Q. Zhang J. (2021). Genome-wide identification, classification and expression analysis of the Hsf and Hsp70 gene families in maize. *Gene* 770:145348. 10.1016/j.gene.2020.145348 33333230

[B118] Jiang C. Zu C. Lu D. Zheng Q. Shen J. Wang H. (2017). Effect of exogenous selenium supply on photosynthesis, Na^+^ accumulation and antioxidative capacity of maize (*Zea mays* L.) under salinity stress. *Sci. Rep.* 7:42039. 10.1038/srep42039 28169318 PMC5294586

[B119] Joshi J. Hasnain G. Logue T. Lynch M. Wu S. Guan J.-C. (2021). A core metabolome response of maize leaves subjected to long-duration abiotic stresses. *Metabolites* 11:797. 10.3390/metabo11110797 34822455 PMC8625080

[B120] Kaplan F. Kopka J. Haskell D. W. Zhao W. Schiller K. C. Gatzke N. (2004). Exploring the temperature-stress metabolome of Arabidopsis. *Plant Physiol.* 136 4159–4168. 10.1104/pp.104.052142 15557093 PMC535846

[B121] Kaspar T. Bland W. L. (1992). Soil temperature and root growth. *Soil Sci.* 154 290–299. 10.1097/00010694-199210000-00005

[B122] Keeling P. Banisadr R. Barone L. Wasserman B. Singletary G. (1994). Effect of temperature on enzymes in the pathway of starch biosynthesis in developing wheat and maize grain. *Funct. Plant Biol.* 21 807–827. 10.1071/pp9940807

[B123] Ketehouli T. Idrice Carther K. F. Noman M. Wang F.-W. Li X.-W. Li H.-Y. (2019). Adaptation of plants to salt stress: characterization of Na+ and K+ transporters and role of CBL gene family in regulating salt stress response. *Agronomy* 9:687. 10.3390/agronomy9110687

[B124] Khaeim H. Kende Z. Jolánkai M. Kovács G. P. Gyuricza C. Tarnawa Á. (2022). Impact of temperature and water on seed germination and seedling growth of maize (*Zea mays* L.). *Agronomy* 12:397. 10.3390/agronomy12020397

[B125] Khajuria P. Singh A. K. Singh R. (2016). Identification of heat stress tolerant genotypes in bread wheat. *Electron. J. Plant Breed.* 7 1446–1452.

[B126] Khakwani A. A. Dennett M. D. Munir M. Baloch M. S. (2012). Wheat yield response to physiological limitations under water stress condition. *J. Anim. Plant Sci.* 22 773–780.

[B127] Khan A. Ali M. Khattak A. M. Gai W.-X. Zhang H.-X. Wei A.-M. (2019). Heat shock proteins: dynamic biomolecules to counter plant biotic and abiotic stresses. *Int. J. Mol. Sci.* 20:5321. 10.3390/ijms20215321 31731530 PMC6862505

[B128] Khan M. I. R. Fatma M. Per T. S. Anjum N. A. Khan N. A. (2015). Salicylic acid-induced abiotic stress tolerance and underlying mechanisms in plants. *Front. Plant Sci.* 6:462. 10.3389/fpls.2015.00462 26175738 PMC4485163

[B129] Kim J.-M. To T. K. Ishida J. Matsui A. Kimura H. Seki M. (2012). Transition of chromatin status during the process of recovery from drought stress in *Arabidopsis thaliana*. *Plant Cell Physiol.* 53 847–856. 10.1093/pcp/pcs053 22505693

[B130] Kimotho R. N. Baillo E. H. Zhang Z. (2019). Transcription factors involved in abiotic stress responses in maize (*Zea mays* L.) and their roles in enhanced productivity in the post genomics era. *PeerJ* 7:e7211. 10.7717/peerj.7211 31328030 PMC6622165

[B131] Kiniry J. Bonhomme R. (1991). Predicting maize phenology. *Predict. Crop Phenol.* 11 115–131.

[B132] Klein R. D. Chidawanyika T. Tims H. S. Meulia T. Bouchard R. A. Pett V. B. (2014). Chaperone function of two small heat shock proteins from maize. *Plant Sci.* 221-222 48–58. 10.1016/j.plantsci.2014.01.012 24656335

[B133] Kong L. Wang F. Si J. Feng B. Zhang B. Li S. (2013). Increasing in ROS levels and callose deposition in peduncle vascular bundles of wheat (*Triticum aestivum* L.) grown under nitrogen deficiency. *J. Plant Interact.* 8 109–116. 10.1080/17429145.2012.712723

[B134] Kørner C. J. Du X. Vollmer M. E. Pajerowska-Mukhtar K. M. (2015). Endoplasmic reticulum stress signaling in plant immunity—at the crossroad of life and death. *Int. J. Mol. Sci.* 16 26582–26598. 10.3390/ijms161125964 26556351 PMC4661823

[B135] Kraus K. Hnilickova H. Pecka J. Lhotska M. Bezdickova A. Martinek P. (2022). The effect of the application of stimulants on the photosynthetic apparatus and the yield of winter wheat. *Agronomy* 12:78. 10.3390/agronomy12010078

[B136] Kulski J. K. (ed.) (2016). “Next-generation sequencing—an overview of the history, tools, and “Omic” applications,” in *Next Generation Sequencing–Advances, Applications and Challenges*, (London: InTech), 3–60.

[B137] Kumar A. Kaushik P. (2021). Heat stress and its impact on plant function: an update. *Preprints* 2021:2021080489.

[B138] Kumar K. Singh I. Aggarwal C. Tewari I. Jha A. K. Yadava P. (2019). Expression profiling of heat shock protein genes in two contrasting maize inbred lines. *Int. J. Curr. Microbiol. Appl. Sci.* 8 347–358. 10.20546/ijcmas.2019.806.039

[B139] Kumar M. Kumar Patel M. Kumar N. Bajpai A. B. Siddique K. H. M. (2021). Metabolomics and molecular approaches reveal drought stress tolerance in plants. *Int. J. Mol. Sci.* 22:9108. 10.3390/ijms22179108 34502020 PMC8431676

[B140] Kumari S. Sabharwal V. P. Kushwaha H. R. Sopory S. K. Singla-Pareek S. L. Pareek A. (2009). Transcriptome map for seedling stage specific salinity stress response indicates a specific set of genes as candidate for saline tolerance in *Oryza sativa* L. *Funct. Integr. Genomics* 9 109–123. 10.1007/s10142-008-0088-5 18594887

[B141] Lafarge T. Bueno C. Frouin J. Jacquin L. Courtois B. Ahmadi N. (2017). Genome-wide association analysis for heat tolerance at flowering detected a large set of genes involved in adaptation to thermal and other stresses. *PLoS One* 12:e0171254. 10.1371/journal.pone.0171254 28152098 PMC5289576

[B142] Le Nguyen K. Grondin A. Courtois B. Gantet P. (2019). Next-generation sequencing accelerates crop gene discovery. *Trends Plant Sci.* 24 263–274. 10.1016/j.tplants.2018.11.008 30573308

[B143] Leng P. Zhao J. (2020). Transcription factors as molecular switches to regulate drought adaptation in maize. *Theor. Appl. Genet.* 133 1455–1465. 10.1007/s00122-019-03494-y 31807836

[B144] Li B. Gao K. Ren H. Tang W. (2018). Molecular mechanisms governing plant responses to high temperatures. *J. Integr. Plant Biol.* 60 757–779. 10.1111/jipb.12701 30030890

[B145] Li Z. Srivastava R. Tang J. Zheng Z. Howell S. H. (2018). *Cis*-effects condition the induction of a major unfolded protein response factor, *ZmbZIP60*, in response to heat stress in maize. *Front. Plant Sci.* 9:833. 10.3389/fpls.2018.00833 30008724 PMC6034121

[B146] Li H.-C. Zhang H.-N. Li G.-L. Liu Z.-H. Zhang Y.-M. Zhang H.-M. (2015). Expression of maize heat shock transcription factor gene ZmHsf06 enhances the thermotolerance and drought-stress tolerance of transgenic Arabidopsis. *Funct. Plant Biol.* 42 1080–1091. 10.1071/FP15080 32480747

[B147] Li Z. G. Xie L. R. Li X. J. (2015). Hydrogen sulfide acts as a downstream signal molecule in salicylic acid-induced heat tolerance in maize (*Zea mays* L.) seedlings. *J. Plant Physiol.* 177 121–127. 10.1016/j.jplph.2014.12.018 25727780

[B148] Li J. Zhang L. Elbaiomy R. G. Chen L. Wang Z. Jiao J. (2022). Evolution analysis of *FRIZZY PANICLE* (*FZP*) orthologs explored the mutations in DNA coding sequences in the grass family (Poaceae). *PeerJ* 10:e12880. 10.7717/peerj.12880 35295554 PMC8919851

[B149] Li J. Zhang L. Yuan Y. Wang Q. Elbaiomy R. Zhou W. (2021). In silico functional prediction and expression analysis of C2H2 zinc-finger family transcription factor revealed regulatory role of ZmZFP126 in maize growth. *Front. Genet.* 12:770427. 10.3389/fgene.2021.770427 34804129 PMC8602080

[B150] Li N. Euring D. Cha J. Y. Lin Z. Lu M. Huang L.-J. (2021). Plant hormone-mediated regulation of heat tolerance in response to global climate change. *Front. Plant Sci.* 11:627969. 10.3389/fpls.2020.627969 33643337 PMC7905216

[B151] Li Y. T. Xu W. W. Ren B. Z. Zhao B. Zhang J. Liu P. (2020). High temperature reduces photosynthesis in maize leaves by damaging chloroplast ultrastructure and photosystem II. *J. Agron. Crop Sci.* 206 548–564. 10.1111/jac.12401

[B152] Li Z. Tang J. Srivastava R. Bassham D. C. Howell S. H. (2020). The transcription factor bZIP60 links the unfolded protein response to the heat stress response in maize. *Plant Cell* 32 3559–3575. 10.1105/tpc.20.00260 32843434 PMC7610289

[B153] Li Z. Howell S. H. (2021). Heat stress responses and thermotolerance in maize. *Int. J. Mol. Sci.* 22:948. 10.3390/ijms22020948 33477941 PMC7833377

[B154] Li Z. G. (2015). Synergistic effect of antioxidant system and osmolyte in hydrogen sulfide and salicylic acid crosstalk-induced heat tolerance in maize (*Zea mays* L.) seedlings. *Plant Signal. Behav.* 10:e1051278. 10.1080/15592324.2015.1051278 26337076 PMC4883857

[B155] Li Z.-G. Ye X.-Y. Qiu X.-M. (2019). Glutamate signaling enhances the heat tolerance of maize seedlings by plant glutamate receptor-like channels-mediated calcium signaling. *Protoplasma* 256 1165–1169. 10.1007/s00709-019-01351-9 30675652

[B156] Lieu E. L. Kelekar N. Bhalla P. Kim J. (2021). Fructose and mannose in inborn errors of metabolism and cancer. *Metabolites* 11:479. 10.3390/metabo11080479 34436420 PMC8397987

[B157] Lima R. B. Dos Santos T. B. Vieira L. G. E. Ferrarese M. D. L. L. Ferrarese-Filho O. Donatti L. (2013). Heat stress causes alterations in the cell-wall polymers and anatomy of coffee leaves (*Coffea arabica* L.). *Carbohydr. Polym.* 93 135–143. 10.1016/j.carbpol.2012.05.015 23465912

[B158] Lin F. Ding H. Wang J. Zhang H. Zhang A. Zhang Y. (2009). Positive feedback regulation of maize NADPH oxidase by mitogen-activated protein kinase cascade in abscisic acid signalling. *J. Exp. Bot.* 60 3221–3238. 10.1093/jxb/erp157 19592501 PMC2718220

[B159] Lin F. Wani S. H. Collins P. J. Wen Z. Li W. Zhang N. (2020). QTL mapping and GWAS for identification of loci conferring partial resistance to Pythium sylvaticum in soybean(*Glycine max* (L.) Merr). *Mol. Breed.* 40:54.

[B160] Lin Y.-X. Jiang H.-Y. Chu Z.-X. Tang X.-L. Zhu S.-W. Cheng B.-J. (2011). Genome-wide identification, classification and analysis of heat shock transcription factor family in maize. *BMC Genomics* 12:76. 10.1186/1471-2164-12-76 21272351 PMC3039612

[B161] Liu J. Niu Y. Zhang J. Zhou Y. Ma Z. Huang X. (2018). Ca2+ channels and Ca2+ signals involved in abiotic stress responses in plant cells: recent advances. *Plant Cell Tissue Organ Cult.* 132 413–424. 10.1007/s11240-017-1350-0

[B162] Liu J.-X. Srivastava R. Che P. Howell S. H. (2007). An endoplasmic reticulum stress response in *Arabidopsis* is mediated by proteolytic processing and nuclear relocation of a membrane-associated transcription factor, bZIP28. *Plant Cell* 19 4111–4119. 10.1105/tpc.106.050021 18156219 PMC2217655

[B163] Liu L. Hao Z. Weng J. Li M. Zhang D. Bai L. (2012). Identification of drought-responsive genes by cDNA-amplified fragment length polymorphism in maize. *Ann. Appl. Biol.* 161 203–213. 10.1111/j.1744-7348.2012.00565.x

[B164] Liu Z. Hubbard K. G. Lin X. Yang X. (2013). Negative effects of climate warming on maize yield are reversed by the changing of sowing date and cultivar selection in Northeast China. *Glob. Change Biol.* 19 3481–3492. 10.1111/gcb.12324 23857749

[B165] Lobell D. B. Bänziger M. Magorokosho C. Vivek B. (2011). Nonlinear heat effects on African maize as evidenced by historical yield trials. *Nat. Clim. Change* 1 42–45. 10.1038/nclimate1043

[B166] Longmei N. Gill G. K. Zaidi P. H. Kumar R. Nair S. K. Hindu V. (2021). Genome wide association mapping for heat tolerance in sub-tropical maize. *BMC Genomics* 22:154. 10.1186/s12864-021-07463-y 33663389 PMC7934507

[B167] Lorenz A. J. Chao S. Asoro F. G. Heffner E. L. Hayashi T. Iwata H. (2011). Genomic selection in plant breeding: knowledge and prospects. *Adv. Agron.* 110 77–123.

[B168] Ludwig-Müller J. (2011). Auxin conjugates: their role for plant development and in the evolution of land plants. *J. Exp. Bot.* 62 1757–1773. 10.1093/jxb/erq412 21307383

[B169] Luna E. Pastor V. Robert J. Flors V. Mauch-Mani B. Ton J. (2011). Callose deposition: a multifaceted plant defense response. *Mol. Plant Microbe Interact.* 24 183–193. 10.1094/MPMI-07-10-0149 20955078

[B170] Ma H. Liu C. Li Z. Ran Q. Xie G. Wang B. (2018). ZmbZIP4 contributes to stress resistance in maize by regulating ABA synthesis and root development. *Plant Physiol.* 178 753–770. 10.1104/pp.18.00436 30126870 PMC6181033

[B171] Ma P. Liu J. Yang X. Ma R. (2013). Genome-wide identification of the maize calcium-dependent protein kinase gene family. *Appl. Biochem. Biotechnol.* 169 2111–2125. 10.1007/s12010-013-0125-2 23397323

[B172] Mafakheri A. Siosemardeh A. Bahramnejad B. Struik P. C. Sohrabi Y. (2010). Effect of drought stress on yield, proline and chlorophyll contents in three chickpea cultivars. *Aust. J. Crop Sci.* 4 580–585.

[B173] Magaña Ugarte R. Escudero A. Gavilán R. G. (2019). Metabolic and physiological responses of Mediterranean high-mountain and alpine plants to combined abiotic stresses. *Physiol. Plant.* 165 403–412. 10.1111/ppl.12898 30536685

[B174] Maitra P. Al-Rashid J. Barman N. C. Khan M. Morshed N. Mandal D. (2021). Sand particle size and phosphorus amount affect *Rhizophagus irregularis* spore production using in vitro propagated spore as a starter inoculum in rhizosphere of maize (*Zea mays*) plantlets. *J. Fungi* 7:846. 10.3390/jof7100846 34682267 PMC8541049

[B175] Majeran W. van Wijk K. J. (2009). Cell-type-specific differentiation of chloroplasts in C4 plants. *Trends Plant Sci.* 14 100–109. 10.1016/j.tplants.2008.11.006 19162526

[B176] Malenica N. Duniæ J. A. Vukadinoviæ L. Cesar V. Šimiæ D. (2021). Genetic approaches to enhance multiple stress tolerance in maize. *Genes* 12:1760. 10.3390/genes12111760 34828366 PMC8617808

[B177] Marcec M. J. Gilroy S. Poovaiah B. W. Tanaka K. (2019). Mutual interplay of Ca2+ and ROS signaling in plant immune response. *Plant Sci.* 283 343–354. 10.1016/j.plantsci.2019.03.004 31128705

[B178] Markelz R. C. Strellner R. S. Leakey A. D. (2011). Impairment of C4 photosynthesis by drought is exacerbated by limiting nitrogen and ameliorated by elevated [CO2] in maize. *J. Exp. Bot.* 62 3235–3246. 10.1093/jxb/err056 21398428

[B179] Marrs K. A. Casey E. S. Capitant S. A. Bouchard R. A. Dietrich P. S. Mettler I. J. (1993). Characterization of two maize HSP90 heat shock protein genes: expression during heat shock, embryogenesis, and pollen development. *Dev. Genet.* 14 27–41. 10.1002/dvg.1020140105 7683257

[B180] McKevith B. (2004). Nutritional aspects of cereals. *Nutr. Bull.* 29 111–142. 10.1111/j.1467-3010.2004.00418.x

[B181] Medeiros D. B. Brotman Y. Fernie A. R. (2021). The utility of metabolomics as a tool to inform maize biology. *Plant Commun.* 2:100187. 10.1016/j.xplc.2021.100187 34327322 PMC8299083

[B182] Meena A. K. Singh D. K. Pandey P. C. Nanda G. (2019). Dynamics of dry matter and nitrogen distribution in transplanted rice on mollisols. *J. Plant Nutr.* 42 749–758. 10.1080/01904167.2019.1567777

[B183] Meena M. K. Ghawana S. Dwivedi V. Roy A. Chattopadhyay D. (2015). Expression of chickpea CIPK25 enhances root growth and tolerance to dehydration and salt stress in transgenic tobacco. *Front. Plant Sci.* 6:683. 10.3389/fpls.2015.00683 26442004 PMC4561800

[B184] Meena R. S. Lal R. Yadav G. S. (2020). Long-term impact of topsoil depth and amendments on carbon and nitrogen budgets in the surface layer of an Alfisol in Central Ohio. *Catena* 194:104752. 10.1016/j.catena.2020.104752

[B185] Miller G. Mittler R. (2006). Could heat shock transcription factors function as hydrogen peroxide sensors in plants? *Ann. Bot.* 98 279–288. 10.1093/aob/mcl107 16740587 PMC2803459

[B186] Mishra V. Cruise J. F. Mecikalski J. R. (2021). Assimilation of coupled microwave/thermal infrared soil moisture profiles into a crop model for robust maize yield estimates over Southeast United States. *Eur. J. Agron.* 123:126208. 10.1016/j.eja.2020.126208

[B187] Mizoi J. Shinozaki K. Yamaguchi-Shinozaki K. (2012). AP2/ERF family transcription factors in plant abiotic stress responses. *Biochim. Biophys. Acta* 1819 86–96. 10.1016/j.bbagrm.2011.08.004 21867785

[B188] Mo F. Sun M. Liu X.-Y. Wang J.-Y. Zhang X.-C. Ma B. (2016). Phenological responses of spring wheat and maize to changes in crop management and rising temperatures from 1992 to 2013 across the Loess Plateau. *Field Crops Res.* 196 337–347. 10.1016/j.fcr.2016.06.024

[B189] Mortimer J. C. Laohavisit A. Macpherson N. Webb A. Brownlee C. Battey N. H. (2008). Annexins: multifunctional components of growth and adaptation. *J. Exp. Bot.* 59 533–544. 10.1093/jxb/erm344 18267940

[B190] Mujahid A. Pumford N. R. Bottje W. Nakagawa K. Miyazawa T. Akiba Y. (2007). Mitochondrial oxidative damage in chicken skeletal muscle induced by acute heat stress. *J. Poult. Sci.* 44 439–445. 10.2141/jpsa.44.439

[B191] Navarro J. A. Saiz-Bonilla M. Sanchez-Navarro J. A. Pallas V. (2021). The mitochondrial and chloroplast dual targeting of a multifunctional plant viral protein modulates chloroplast-to-nucleus communication, RNA silencing suppressor activity, encapsidation, pathogenesis and tissue tropism. *Plant J.* 108 197–218. 10.1111/tpj.15435 34309112

[B192] Naveed S. Aslam M. Maqbool M. Bano S. Zaman Q. Ahmad R. (2014). Physiology of high temperature stress tolerance at reproductive stages in maize. *J. Anim. Plant Sci.* 24 1141–1145.

[B193] Nawkar G. M. Lee E. S. Shelake R. M. Park J. H. Ryu S. W. Kang C. H. (2018). Activation of the transducers of unfolded protein response in plants. *Front. Plant Sci.* 9:214. 10.3389/fpls.2018.00214 29515614 PMC5826264

[B194] Neill E. M. Byrd M. C. Billman T. Brandizzi F. Stapleton A. E. (2019). Plant growth regulators interact with elevated temperature to alter heat stress signaling via the Unfolded Protein Response in maize. *Sci. Rep.* 9:10392. 10.1038/s41598-019-46839-9 31316112 PMC6637120

[B195] Nestler J. Liu S. Wen T.-J. Paschold A. Marcon C. Tang H. M. (2014). Roothairless5, which functions in maize (*Zea mays* L.) root hair initiation and elongation encodes a monocot-specific NADPH oxidase. *Plant J.* 79 729–740. 10.1111/tpj.12578 24902980

[B196] Nichols C. (2005). *Functional Characterisation of Plant Annexins*. Ph.D. thesis. Cambridge: University of Cambridge.

[B197] Nieto-Sotelo J. Martínez L. M. Ponce G. Cassab G. I. Alagón A. Meeley R. B. (2002). Maize HSP101 plays important roles in both induced and basal thermotolerance and primary root growth. *Plant Cell* 14 1621–1633. 10.1105/tpc.010487 12119379 PMC150711

[B198] Nieto-Sotelo J. Vierling E. Ho T.-H. D. (1990). Cloning, sequence analysis, and expression of a cDNA encoding a plastid-localized heat shock protein in maize. *Plant Physiol.* 93 1321–1328. 10.1104/pp.93.4.1321 16667620 PMC1062675

[B199] Nievola C. C. Carvalho C. P. Carvalho V. Rodrigues E. (2017). Rapid responses of plants to temperature changes. *Temperature* 4 371–405. 10.1080/23328940.2017.1377812 29435478 PMC5800372

[B200] Nijabat A. Bolton A. Mahmood-Ur-Rehman M. Shah A. I. Hussain R. Naveed N. H. (2020). Cell membrane stability and relative cell injury in response to heat stress during early and late seedling stages of diverse carrot (*Daucus carota* L.) germplasm. *Hortscience* 55 1446–1452. 10.21273/hortsci15058-20

[B201] Niu S. Du X. Wei D. Liu S. Tang Q. Bian D. (2021). Heat stress after pollination reduces kernel number in maize by insufficient assimilates. *Front. Genet.* 12:728166. 10.3389/fgene.2021.728166 34691151 PMC8532994

[B202] Niu Y. Xiang Y. (2018). An overview of biomembrane functions in plant responses to high-temperature stress. *Front. Plant Sci.* 9:915. 10.3389/fpls.2018.00915 30018629 PMC6037897

[B203] Ohama N. Sato H. Shinozaki K. Yamaguchi-Shinozaki K. (2017). Transcriptional regulatory network of plant heat stress response. *Trends Plant Sci.* 22 53–65. 10.1016/j.tplants.2016.08.015 27666516

[B204] O’Leary B. Park J. Plaxton W. C. (2011). The remarkable diversity of plant PEPC (phosphoenolpyruvate carboxylase): recent insights into the physiological functions and post-translational controls of non-photosynthetic PEPCs. *Biochem. J.* 436 15–34. 10.1042/BJ20110078 21524275

[B205] Osuna D. Prieto P. Aguilar M. (2015). Control of seed germination and plant development by carbon and nitrogen availability. *Front. Plant Sci.* 6:1023. 10.3389/fpls.2015.01023 26635847 PMC4649081

[B206] Pamplona R. (2008). Membrane phospholipids, lipoxidative damage and molecular integrity: a causal role in aging and longevity. *Biochim. Biophys. Acta* 1777 1249–1262. 10.1016/j.bbabio.2008.07.003 18721793

[B207] Pandey N. Ranjan A. Pant P. Tripathi R. K. Ateek F. Pandey H. P. (2013). CAMTA 1 regulates drought responses in *Arabidopsis thaliana*. *BMC Genomics* 14:216. 10.1186/1471-2164-14-216 23547968 PMC3621073

[B208] Pandey R. Maranville J. Admou A. (2000). Deficit irrigation and nitrogen effects on maize in a Sahelian environment: I. Grain yield and yield components. *Agric. Water Manag.* 46 15–27. 10.1016/s0378-3774(00)00074-3

[B209] Parmar A. Sturm B. Hensel O. (2017). Crops that feed the world: production and improvement of cassava for food, feed, and industrial uses. *Food Secur.* 9 907–927. 10.1007/s12571-017-0717-8

[B210] Pastor-Cantizano N. Ko D. K. Angelos E. Pu Y. Brandizzi F. (2020). Functional diversification of ER stress responses in *Arabidopsis*. *Trends Biochem. Sci.* 45 123–136. 10.1016/j.tibs.2019.10.008 31753702 PMC6980780

[B211] Paul E. A. Paustian K. H. Elliott E. Cole C. V. (1996). *Soil Organic Matter in Temperate AgroecosystemsLong Term Experiments in North America.* Boca Raton, FL: CRC Press.

[B212] Pegoraro C. Mertz L. M. Da Maia L. C. Rombaldi C. V. De Oliveira A. C. (2011). Importance of heat shock proteins in maize. *J. Crop Sci. Biotechnol.* 14 85–95.

[B213] Popp J. Petö K. Nagy J. (2013). Pesticide productivity and food security. A review. *Agron. Sustain. Dev.* 33 243–255. 10.1007/s13593-012-0105-x

[B214] Prasad T. Hack E. Hallberg R. (1990). Function of the maize mitochondrial chaperonin hsp60: specific association between hsp60 and newly synthesized F1-ATPase alpha subunits. *Mol. Cell. Biol.* 10 3979–3986. 10.1128/mcb.10.8.3979-3986.1990 1973526 PMC360908

[B215] Qian Y. Ren Q. Zhang J. Chen L. (2019). Transcriptomic analysis of the maize (*Zea mays* L.) inbred line B73 response to heat stress at the seedling stage. *Gene* 692 68–78. 10.1016/j.gene.2018.12.062 30641208

[B216] Qin F. Kakimoto M. Sakuma Y. Maruyama K. Osakabe Y. Tran L. S. P. (2007). Regulation and functional analysis of ZmDREB2A in response to drought and heat stresses in *Zea mays* L. *Plant J.* 50 54–69. 10.1111/j.1365-313X.2007.03034.x 17346263

[B217] Rahman M. A. Chikushi J. Yoshida S. Karim A. (2009). Growth and yield components of wheat genotypes exposed to high temperature stress under control environment. *Bangladesh J. Agric. Res.* 34 360–372. 10.3329/bjar.v34i3.3961

[B218] Ranum P. Peña-Rosas J. P. Garcia-Casal M. N. (2014). Global maize production, utilization, and consumption. *Ann. N. Y. Acad. Sci.* 1312 105–112. 10.1111/nyas.12396 24650320

[B219] Rashed M. A.-S. Abou-Deif M. H. Khalil K. M. Mahmoud F. E.-S. (2021). Expression levels of heat shock proteins through western blot and real-time polymerase chain reaction in maize. *Jordan J. Biol. Sci.* 14 671–676. 10.1007/s00726-010-0538-y 20213438

[B220] Razzaq A. Kaur P. Akhter N. Wani S. H. Saleem F. (2021). Next-generation breeding strategies for climate-ready crops. *Front. Plant Sci.* 12:620420. 10.3389/fpls.2021.620420 34367194 PMC8336580

[B221] Reddy A. Reddy V. S. Golovkin M. (2000). A calmodulin binding protein from *Arabidopsis* is induced by ethylene and contains a DNA-binding motif. *Biochem. Biophys. Res. Commun.* 279 762–769. 10.1006/bbrc.2000.4032 11162426

[B222] Reddy A. S. Ben-Hur A. Day I. S. (2011). Experimental and computational approaches for the study of calmodulin interactions. *Phytochemistry* 72 1007–1019. 10.1016/j.phytochem.2010.12.022 21338992

[B223] Reddy P. S. Kavi Kishor P. B. Seiler C. Kuhlmann M. Eschen-Lippold L. Lee J. (2014). Unraveling regulation of the small heat shock proteins by the heat shock factor *HvHsfB2c* in barley: its implications in drought stress response and seed development. *PLoS One* 9:e89125. 10.1371/journal.pone.0089125 24594978 PMC3942355

[B224] Reddy V. S. Day I. S. Thomas T. Reddy A. S. N. (2004). KIC, a novel Ca2+ binding protein with one EF-hand motif, interacts with a microtubule motor protein and regulates trichome morphogenesis. *Plant Cell* 16 185–200. 10.1105/tpc.016600 14688294 PMC301404

[B225] Reimold A. M. Etkin A. Clauss I. Perkins A. Friend D. S. Zhang J. (2000). An essential role in liver development for transcription factor XBP-1. *Genes Dev.* 14 152–157. 10.1101/gad.14.2.152 10652269 PMC316338

[B226] Rezaei E. E. Webber H. Gaiser T. Naab J. Ewert F. (2015). Heat stress in cereals: mechanisms and modelling. *Eur. J. Agron.* 64 98–113. 10.1016/j.eja.2014.10.003

[B227] Ribeiro C. Hennen-Bierwagen T. A. Myers A. M. Cline K. Settles A. M. (2020). Engineering 6-phosphogluconate dehydrogenase to improve heat tolerance in maize seed development. *bioRxiv [Preprint]* 10.1101/2020.05.21.108985 PMC7776907 33323483

[B228] Rochaix J.-D. (2011). Regulation of photosynthetic electron transport. *Biochim. Biophys. Acta* 1807 375–383. 21118674 10.1016/j.bbabio.2010.11.010

[B229] Rodziewicz P. Swarcewicz B. Chmielewska K. Wojakowska A. Stobiecki M. (2014). Influence of abiotic stresses on plant proteome and metabolome changes. *Acta Physiol. Plant.* 36 1–19. 10.1007/s11738-013-1402-y

[B230] Rooney W. L. Blumenthal J. Bean B. Mullet J. E. (2007). Designing sorghum as a dedicated bioenergy feedstock. *Biofuels Bioprod. Biorefin.* 1 147–157. 10.1186/s13068-017-0892-z 28878821 PMC5584014

[B231] Rosenzweig C. Iglesius A. Yang X.-B. Epstein P. R. Chivian E. (2001). Climate change and extreme weather events: implications for food production, plant diseases, and pests. *Glob. Change Hum. Health* 2 90–104.

[B232] Ruan Y.-L. Jin Y. Yang Y.-J. Li G.-J. Boyer J. S. (2010). Sugar input, metabolism, and signaling mediated by invertase: roles in development, yield potential, and response to drought and heat. *Mol. Plant* 3 942–955. 10.1093/mp/ssq044 20729475

[B233] Rupwate S. D. Rajasekharan R. (2012). Plant phosphoinositide-specific phospholipase C: an insight. *Plant Signal. Behav.* 7 1281–1283. 10.4161/psb.21436 22902702 PMC3493414

[B234] Ryel R. Caldwell M. Yoder C. Or D. Leffler A. (2002). Hydraulic redistribution in a stand of *Artemisia tridentata*: evaluation of benefits to transpiration assessed with a simulation model. *Oecologia* 130 173–184. 10.1007/s004420100794 28547139

[B235] Sabagh A. E. Hossain A. Iqbal M. A. Barutçular C. Islam M. S. Çiǧ F. (2020). “Maize adaptability to heat stress under changing climate,” in *Plant Stress Physiology*, ed. Hossain A. (London: IntechOpen).

[B236] Sachs M. M. Subbaiah C. C. Saab I. N. (1996). Anaerobic gene expression and flooding tolerance in maize. *J. Exp. Bot.* 47 1–15. 10.1104/pp.109.2.433 7480340 PMC157605

[B237] Sagi M. Fluhr R. (2006). Production of reactive oxygen species by plant NADPH oxidases. *Plant Physiol.* 141 336–340. 10.1104/pp.106.078089 16760484 PMC1475462

[B238] Sah R. Chakraborty M. Prasad K. Pandit M. Tudu V. Chakravarty M. (2020). Impact of water deficit stress in maize: phenology and yield components. *Sci. Rep.* 10:2944. 10.1038/s41598-020-59689-7 32076012 PMC7031221

[B239] Sakai A. Larcher W. (2012). *Frost Survival of Plants: Responses and Adaptation to Freezing Stress.* Berlin: Springer Science & Business Media.

[B240] Sallam A. Amro A. El-Akhdar A. Dawood M. F. A. Kumamaru T. Stephen Baenziger P. (2018). Genetic diversity and genetic variation in morpho-physiological traits to improve heat tolerance in Spring barley. *Mol. Biol. Rep.* 45 2441–2453. 10.1007/s11033-018-4410-6 30411192

[B241] Sánchez B. Rasmussen A. Porter J. R. (2014). Temperatures and the growth and development of maize and rice: a review. *Glob. Change Biol.* 20 408–417. 10.1111/gcb.12389 24038930

[B242] Sang J. Zhang A. Lin F. Tan M. Jiang M. (2008). Cross-talk between calcium-calmodulin and nitric oxide in abscisic acid signaling in leaves of maize plants. *Cell Res.* 18 577–588. 10.1038/cr.2008.39 18364679

[B243] Schramm F. Larkindale J. Kiehlmann E. Ganguli A. Englich G. Vierling E. (2008). A cascade of transcription factor DREB2A and heat stress transcription factor HsfA3 regulates the heat stress response of *Arabidopsis*. *Plant J.* 53 264–274. 10.1111/j.1365-313X.2007.03334.x 17999647

[B244] Seetharam K. Kuchanur P. H. Koirala K. Tripathi M. P. Patil A. Sudarsanam V. (2021). Genomic regions associated with heat stress tolerance in tropical maize (*Zea mays* L.). *Sci. Rep.* 11:13730. 10.1038/s41598-021-93061-7 34215789 PMC8253795

[B245] Sengupta S. Mukherjee S. Basak P. Majumder A. L. (2015). Significance of galactinol and raffinose family oligosaccharide synthesis in plants. *Front. Plant Sci.* 6:656. 10.3389/fpls.2015.00656 26379684 PMC4549555

[B246] Sewelam N. Jaspert N. Van Der Kelen K. Tognetti V. B. Schmitz J. Frerigmann H. (2014). Spatial H2O2 signaling specificity: H2O2 from chloroplasts and peroxisomes modulates the plant transcriptome differentially. *Mol. Plant* 7 1191–1210. 10.1093/mp/ssu070 24908268

[B247] Sharma A. Kumar V. Shahzad B. Ramakrishnan M. Singh Sidhu G. P. Bali A. S. (2020). Photosynthetic response of plants under different abiotic stresses: a review. *J. Plant Growth Regul.* 39 509–531.

[B248] Sharma A. Shahzad B. Kumar V. Kohli S. K. Sidhu G. P. S. Bali A. S. (2019). Phytohormones regulate accumulation of osmolytes under abiotic stress. *Biomolecules* 9:285. 10.3390/biom9070285 31319576 PMC6680914

[B249] Sharma D. K. Torp A. M. Rosenqvist E. Ottosen C.-O. Andersen S. B. (2017). QTLs and potential candidate genes for heat stress tolerance identified from the mapping populations specifically segregating for *F*v/*F*m in wheat. *Front. Plant Sci.* 8:1668. 10.3389/fpls.2017.01668 29021798 PMC5623722

[B250] Shi S. Li S. Asim M. Mao J. Xu D. Ullah Z. (2018). The *Arabidopsis* calcium-dependent protein kinases (CDPKs) and their roles in plant growth regulation and abiotic stress responses. *Int. J. Mol. Sci.* 19:1900. 10.3390/ijms19071900 29958430 PMC6073581

[B251] Siebers M. H. Slattery R. A. Yendrek C. R. Locke A. M. Drag D. Ainsworth E. A. (2017). Simulated heat waves during maize reproductive stages alter reproductive growth but have no lasting effect when applied during vegetative stages. *Agric. Ecosyst. Environ.* 240 162–170. 10.1016/j.agee.2016.11.008

[B252] Silim S. Coe R. Omanga P. Gwata E. (2006). The response of pigeonpea genotypes of different duration types to variation in temperature and photoperiod under field conditions in Kenya. *J. Food Agric. Environ.* 4 209–214.

[B253] Singh R. Gupta R. Bhardwaj R. Singh R. (2021). “CRISPR/CAS9 technologies to enhance tolerance to abiotic stress in crop plants,” in *Environmental Stress Physiology of Plants and Crop Productivity*, eds Kaur T. Arora S. (New York, NY: Bantam Books), 206. 10.2174/9781681087900121010016

[B254] Singletary G. W. Banisadr R. Keeling P. L. (1994). Heat stress during grain filling in maize: effects on carbohydrate storage and metabolism. *Funct. Plant Biol.* 21 829–841. 10.1071/pp9940829

[B255] Slama I. Abdelly C. Bouchereau A. Flowers T. Savouré A. (2015). Diversity, distribution and roles of osmoprotective compounds accumulated in halophytes under abiotic stress. *Ann. Bot.* 115 433–447. 10.1093/aob/mcu239 25564467 PMC4332610

[B256] Song J. Weng Q. Ma H. Yuan J. Wang L. Liu Y. (2016). Cloning and expression analysis of the Hsp70 gene ZmERD2 in *Zea mays*. *Biotechnol. Biotechnol. Equip.* 30 219–226. 10.1080/13102818.2015.1131625

[B257] Song X. Weng Q. Zhao Y. Ma H. Song J. Su L. (2018). Cloning and expression analysis of ZmERD3 gene from *Zea mays*. *Iran J. Biotechnol.* 16:e1593. 10.21859/ijb.1593 30805385 PMC6371631

[B258] Stanley D. W. Parkin K. L. (1991). Biological membrane deterioration and associated quality losses in food tissues. *Crit. Rev. Food Sci. Nutr.* 30 487–553. 10.1080/10408399109527554 1958293

[B259] Stavridou E. Voulgari G. Michailidis M. Kostas S. Chronopoulou E. G. Labrou N. E. (2021). Overexpression of a biotic stress-inducible *Pvgstu* gene activates early protective responses in tobacco under combined heat and drought. *Int. J. Mol. Sci.* 22:2352. 10.3390/ijms22052352 33652971 PMC7956764

[B260] Strasser R. J. Tsimilli-Michael M. Srivastava A. (2004). “Analysis of the chlorophyll a fluorescence transient,” in *Chlorophyll a Fluorescence*, eds Papageorgiou G. C. Govindjee (Dordrecht: Springer), 321–362. 10.1007/978-1-4020-3218-9_12

[B261] Su H. Cao Y. Ku L. Yao W. Cao Y. Ren Z. (2018). Dual functions of ZmNF-YA3 in photoperiod-dependent flowering and abiotic stress responses in maize. *J. Exp. Bot.* 69 5177–5189. 10.1093/jxb/ery299 30137393

[B262] Sun L. Liu Y. Kong X. Zhang D. Pan J. Zhou Y. (2012). ZmHSP16.9, a cytosolic class I small heat shock protein in maize (*Zea mays*), confers heat tolerance in transgenic tobacco. *Plant Cell Rep.* 31 1473–1484. 10.1007/s00299-012-1262-8 22534681

[B263] Swapna S. Shylaraj K. S. (2017). Screening for osmotic stress responses in rice varieties under drought condition. *Rice Sci.* 24 1446–1452.

[B264] Takemoto D. Tanaka A. Scott B. (2007). NADPH oxidases in fungi: diverse roles of reactive oxygen species in fungal cellular differentiation. *Fungal Genet. Biol.* 44 1065–1076. 10.1016/j.fgb.2007.04.011 17560148

[B265] Tan Y.-Q. Yang Y. Zhang A. Fei C.-F. Gu L.-L. Sun S.-J. (2020). Three CNGC family members, CNGC5, CNGC6, and CNGC9, are required for constitutive growth of *Arabidopsis* root hairs as Ca2+-permeable channels. *Plant Commun.* 1:100001. 10.1016/j.xplc.2019.100001 33404548 PMC7748020

[B266] Tao F. Zhang Z. (2010). Adaptation of maize production to climate change in North China Plain: quantify the relative contributions of adaptation options. *Eur. J. Agron.* 33 103–116. 10.1016/j.eja.2010.04.002

[B267] Tebaldi C. Lobell D. (2018). Estimated impacts of emission reductions on wheat and maize crops. *Clim. Change* 146 533–545. 10.1007/s10584-015-1537-5

[B268] Tiwari Y. K. Yadav S. K. (2019). High temperature stress tolerance in maize (*Zea mays* L.): physiological and molecular mechanisms. *J. Plant Biol.* 62 93–102. 10.1007/s12374-018-0350-x

[B269] Török Z. Crul T. Maresca B. Schütz G. J. Viana F. Dindia L. (2014). Plasma membranes as heat stress sensors: from lipid-controlled molecular switches to therapeutic applications. *Biochim. Biophys. Acta* 1838 1594–1618. 10.1016/j.bbamem.2013.12.015 24374314

[B270] Tóth S. Z. Nagy V. Puthur J. T. Kovács L. Garab G. (2011). The physiological role of ascorbate as photosystem ii electron donor: protection against photoinactivation in heat-stressed leaves. *Plant Physiol.* 156 382–392. 10.1104/pp.110.171918 21357184 PMC3091034

[B271] Towil L. E. (2010). Long-term pollen storage. *Plant Breed. Rev.* 13 179–207. 10.1002/9780470650059.ch5

[B272] Urquhart W. Chin K. Ung H. Moeder W. Yoshioka K. (2011). The cyclic nucleotide-gated channels AtCNGC11 and 12 are involved in multiple Ca^2+^-dependent physiological responses and act in a synergistic manner. *J. Exp. Bot.* 62 3671–3682. 10.1093/jxb/err074 21414958 PMC3130183

[B273] Van Inghelandt D. Frey F. P. Ries D. Stich B. (2019). QTL mapping and genome-wide prediction of heat tolerance in multiple connected populations of temperate maize. *Sci. Rep.* 9:14418. 10.1038/s41598-019-50853-2 31594984 PMC6783442

[B274] Vardhini B. V. Anjum N. A. (2015). Brassinosteroids make plant life easier under abiotic stresses mainly by modulating major components of antioxidant defense system. *Front. Environ. Sci.* 2:67. 10.3389/fenvs.2014.00067

[B275] Vass I. Cser K. (2009). Janus-faced charge recombinations in photosystem II photoinhibition. *Trends Plant Sci.* 14 200–205. 10.1016/j.tplants.2009.01.009 19303349

[B276] Vitale A. Boston R. S. (2008). Endoplasmic reticulum quality control and the unfolded protein response: insights from plants. *Traffic* 9 1581–1588. 10.1111/j.1600-0854.2008.00780.x 18557840

[B277] von Caemmerer S. Furbank R. T. (2016). Strategies for improving C4 photosynthesis. *Curr. Opin. Plant Biol.* 31 125–134. 10.1016/j.pbi.2016.04.003 27127850

[B278] Wadhwa N. Mathew B. B. Jatawa S. Tiwari A. (2012). Lipid peroxidation: mechanism, models and significance. *Int. J. Curr. Sci.* 3 29–38.

[B279] Wahid A. Gelani S. Asharf M. Foolad M. R. (2007). Heat tolerance in plants: an overview. *Environ. Exp. Bot.* 61 199–223. 10.1016/j.envexpbot.2007.05.011

[B280] Wang C.-T. Ru J.-N. Liu Y.-W. Li M. Zhao D. Yang J.-F. (2018a). Maize WRKY transcription factor ZmWRKY106 confers drought and heat tolerance in transgenic plants. *Int. J. Mol. Sci.* 19:3046. 10.3390/ijms19103046 30301220 PMC6213049

[B281] Wang C.-T. Ru J.-N. Liu Y.-W. Yang J.-F. Li M. Xu Z.-S. (2018b). The maize WRKY transcription factor ZmWRKY40 confers drought resistance in transgenic *Arabidopsis*. *Int. J. Mol. Sci.* 19:2580. 10.3390/ijms19092580 30200246 PMC6164628

[B282] Wang W. Vinocur B. Shoseyov O. Altman A. (2004). Role of plant heat-shock proteins and molecular chaperones in the abiotic stress response. *Trends Plant Sci.* 9 244–252. 10.1016/j.tplants.2004.03.006 15130550

[B283] Wang X. Wang H. Liu S. Ferjani A. Li J. Yan J. (2016). Genetic variation in ZmVPP1 contributes to drought tolerance in maize seedlings. *Nat. Genet.* 48 1233–1241. 10.1038/ng.3636 27526320

[B284] Waqas M. A. Wang X. Zafar S. A. Noor M. A. Hussain H. A. Azher Nawaz M. (2021). Thermal stresses in maize: effects and management strategies. *Plants* 10:293. 10.3390/plants10020293 33557079 PMC7913793

[B285] Waraich E. Ahmad R. Halim A. Aziz T. (2012). Alleviation of temperature stress by nutrient management in crop plants: a review. *J. Soil Sci. Plant Nutr.* 12 221–244. 10.4014/jmb.2105.05009 34226402 PMC9706007

[B286] Weckwerth P. Ehlert B. Romeis T. (2015). ZmCPK1, a calcium-independent kinase member of the *Zea mays* CDPK gene family, functions as a negative regulator in cold stress signalling. *Plant Cell Environ.* 38 544–558. 10.1111/pce.12414 25052912

[B287] Wen W. Li D. Li X. Gao Y. Li W. Li H. (2014). Metabolome-based genome-wide association study of maize kernel leads to novel biochemical insights. *Nat. Commun.* 5:3438. 10.1038/ncomms4438 24633423 PMC3959190

[B288] Wilson D. Muchow R. Murgatroyd C. (1995). Model analysis of temperature and solar radiation limitations to maize potential productivity in a cool climate. *Field Crops Res.* 43 1–18. 10.1016/0378-4290(95)00037-q

[B289] Xalxo R. Yadu B. Chandra J. Chandrakar V. Keshavkant S. (2020). “Alteration in carbohydrate metabolism modulates thermotolerance of plant under heat stress,” in *Heat Stress Tolerance in Plants: Physiological, Molecular and Genetic Perspectives*, eds Wani S. H. Kumar V. (Hoboken, NJ: Wiley), 77–115. 10.1016/j.jprot.2020.103968

[B290] Xu Y. Chu C. Yao S. (2021). The impact of high-temperature stress on rice: challenges and solutions. *Crop J.* 9 963–976. 10.3390/plants10010043 33375473 PMC7823633

[B291] Yadav S. Tiwari Y. K. Singh V. Patil A. A. Shanker A. Lakshmi N. J. (2018). Physiological and biochemical basis of extended and sudden heat stress tolerance in maize. *Proc. Natl. Acad. Sci. India Sect. B Biol. Sci.* 88 249–263. 10.1007/s40011-016-0752-9

[B292] Yadav S. Vanaja M. Maheswari M. (2017). Exogenous application of bio-regulators for alleviation of heat stress in seedlings of maize. *J. Agric. Res.* 2:000137.

[B293] Yamaguchi S. (2008). Gibberellin metabolism and its regulation. *Annu. Rev. Plant Biol.* 59 225–251. 10.1146/annurev.arplant.59.032607.092804 18173378

[B294] Yan K. Chen P. Shao H. Shao C. Zhao S. Brestic M. (2013). Dissection of photosynthetic electron transport process in sweet sorghum under heat stress. *PLoS One* 8:e62100. 10.1371/journal.pone.0062100 23717388 PMC3663741

[B295] Yan Q. Huang Q. Chen J. Li J. Liu Z. Yang Y. (2017). *SYTA* has positive effects on the heat resistance of *Arabidopsis*. *Plant Growth Regul.* 81 467–476. 10.1007/s10725-016-0224-5

[B296] Yan Q. Zong X. Wu F. Li J. Ma T. Zhao Y. (2020). Integrated analysis of co-expression, conserved genes and gene families reveal core regulatory network of heat stress response in *Cleistogenes songorica*, a xerophyte perennial desert plant. *BMC Genomics* 21:715. 10.1186/s12864-020-07122-8 33066732 PMC7566159

[B297] Yang G. Rhodes D. Joly R. J. (1996). Effects of high temperature on membrane stability and chlorophyll fluorescence in glycinebetaine-deficient and glycinebetaine-containing maize lines. *Funct. Plant Biol.* 23 437–443. 10.1071/pp9960437

[B298] Yang L. Wang Y. Yang K. (2021). *Klebsiella variicola* improves the antioxidant ability of maize seedlings under saline-alkali stress. *PeerJ* 9:e11963. 10.7717/peerj.11963 34434665 PMC8359796

[B299] Yang T. Peng H. Whitaker B. D. Jurick W. M. (2013). Differential expression of calcium/calmodulin-regulated SlSRs in response to abiotic and biotic stresses in tomato fruit. *Physiol. Plant.* 148 445–455. 10.1111/ppl.12027 23368882

[B300] Yang T. Poovaiah B. (2002). A calmodulin-binding/CGCG box DNA-binding protein family involved in multiple signaling pathways in plants. *J. Biol. Chem.* 277 45049–45058. 10.1074/jbc.M207941200 12218065

[B301] Yang W. Kong Z. Omo-Ikerodah E. Xu W. Li Q. Xue Y. (2008). Calcineurin B-like interacting protein kinase OsCIPK23 functions in pollination and drought stress responses in rice (*Oryza sativa* L.). *J. Genet. Genomics* 35 531–543, S1–S2. 10.1016/S1673-8527(08)60073-9 18804072

[B302] Yeh S.-Y. Lin H.-H. Chang Y.-M. Chang Y.-L. Chang C.-K. Huang Y.-C. (2021). Maize Golden2-like transcription factors boost rice chloroplast development, photosynthesis, and grain yield. *Plant Physiol.* 188 442–459. 10.1093/plphys/kiab511 34747472 PMC9049120

[B303] Younis A. Ramzan F. Ramzan Y. Zulfiqar F. Ahsan M. Lim K. B. (2020). Molecular markers improve abiotic stress tolerance in crops: a review. *Plants* 9:1374. 10.3390/plants9101374 33076554 PMC7602808

[B304] Yuan B. Z. Sun J. Nishiyama S. (2004). Effect of drip irrigation on strawberry growth and yield inside a plastic greenhouse. *Biosyst. Eng.* 87 237–245. 10.1016/j.biosystemseng.2003.10.014

[B305] Yue R. Lu C. Sun T. Peng T. Han X. Qi J. (2015). Identification and expression profiling analysis of calmodulin-binding transcription activator genes in maize (*Zea mays* L.) under abiotic and biotic stresses. *Front. Plant Sci.* 6:576. 10.3389/fpls.2015.00576 26284092 PMC4516887

[B306] Zhai S. Gao Q. Liu X. Sui Z. Zhang J. (2013). Overexpression of a *Zea mays* phospholipase C1 gene enhances drought tolerance in tobacco in part by maintaining stability in the membrane lipid composition. *Plant Cell Tissue Organ Cult.* 115 253–262. 10.1007/s11240-013-0358-3

[B307] Zhang D. Li Q. Yamori W. Wei M. (2020a). *The Rate-Limiting Step for Photosynthetic CO2 Utilization Under Varying Atmospheric Evaporative Demand in Solanum lycopersicum (Tomato).* Durham, NC: Research Square Company.

[B308] Zhang H. Li G. Fu C. Duan S. Hu D. Guo X. (2020b). Genome-wide identification, transcriptome analysis and alternative splicing events of Hsf family genes in maize. *Sci. Rep.* 10:8073. 10.1038/s41598-020-65068-z 32415117 PMC7229205

[B309] Zhang H. Li G. Hu D. Zhang Y. Zhang Y. Shao H. (2020c). Functional characterization of maize heat shock transcription factor gene ZmHsf01 in thermotolerance. *PeerJ* 8:e8926. 10.7717/peerj.8926 32309048 PMC7153558

[B310] Zhang S.-S. Yang H. Ding L. Song Z.-T. Ma H. Chang F. (2017). Tissue-specific transcriptomics reveals an important role of the unfolded protein response in maintaining fertility upon heat stress in Arabidopsis. *Plant Cell* 29 1007–1023. 10.1105/tpc.16.00916 28442596 PMC5466030

[B311] Zhao C. Liu B. Piao S. Wang X. Lobell D. B. Huang Y. (2017). Temperature increase reduces global yields of major crops in four independent estimates. *Proc. Natl. Acad. Sci. U.S.A.* 114 9326–9331. 10.1073/pnas.1701762114 28811375 PMC5584412

[B312] Zhao J. Lu Z. Wang L. Jin B. (2021). Plant responses to heat stress: physiology, transcription, noncoding RNAs, and epigenetics. *Int. J. Mol. Sci.* 22:117. 10.3390/ijms22010117 33374376 PMC7795586

[B313] Zhao Y. Du H. Wang Y. Wang H. Yang S. Li C. (2021). The calcium-dependent protein kinase ZmCDPK7 functions in heat-stress tolerance in maize. *J. Integr. Plant Biol.* 63 510–527. 10.1111/jipb.13056 33331695

[B314] Zhou L. Lan W. Chen B. Fang W. Luan S. (2015). A calcium sensor-regulated protein kinase, CALCINEURIN B-LIKE PROTEIN-INTERACTING PROTEIN KINASE19, is required for pollen tube growth and polarity. *Plant Physiol.* 167 1351–1360. 10.1104/pp.114.256065 25713341 PMC4378171

[B315] Zhou M.-L. Tang Y.-X. Wu Y.-M. (2012). Genome-wide analysis of AP2/ERF transcription factor family in *Zea mays*. *Curr. Bioinformatics* 7 324–332. 10.2174/157489312802460776

[B316] Zhou Z. H. Wang Y. Ye X. Y. Li Z. G. (2018). Signaling molecule hydrogen sulfide improves seed germination and seedling growth of maize (*Zea mays* L.) under high temperature by inducing antioxidant system and osmolyte biosynthesis. *Front. Plant Sci.* 9:1288. 10.3389/fpls.2018.01288 30233625 PMC6131983

[B317] Zivcak M. Brestic M. Kunderlikova K. Sytar O. Allakhverdiev S. I. (2015). Repetitive light pulse-induced photoinhibition of photosystem I severely affects CO 2 assimilation and photoprotection in wheat leaves. *Photosynth. Res.* 126 449–463. 10.1007/s11120-015-0121-1 25829027

[B318] Zulfiqar F. Ashraf M. (2021). Bioregulators: unlocking their potential role in regulation of the plant oxidative defense system. *Plant Mol. Biol.* 105 11–41. 10.1007/s11103-020-01077-w 32990920

